# Combining *NGN2* Programming with Developmental Patterning Generates Human Excitatory Neurons with NMDAR-Mediated Synaptic Transmission

**DOI:** 10.1016/j.celrep.2018.04.066

**Published:** 2018-05-22

**Authors:** Ralda Nehme, Emanuela Zuccaro, Sulagna Dia Ghosh, Chenchen Li, John L. Sherwood, Olli Pietilainen, Lindy E. Barrett, Francesco Limone, Kathleen A. Worringer, Sravya Kommineni, Ying Zang, Davide Cacchiarelli, Alex Meissner, Rolf Adolfsson, Stephen Haggarty, Jon Madison, Matthias Muller, Paola Arlotta, Zhanyan Fu, Guoping Feng, Kevin Eggan

**Affiliations:** 1 Stanley Center for Psychiatric Research, Broad Institute of Harvard and MIT, Cambridge, MA 02142, USA; 2 Department of Stem Cell and Regenerative Biology, Harvard University, Cambridge, MA 02138, USA; 3 Novartis Institutes for Biomedical Research, Novartis, Cambridge, MA 02139, USA; 4 Umea University, Faculty of Medicine, Department of Clinical Sciences, Psychiatry, 901 85 Umea, Sweden; 5 Center for Human Genetic Research, Massachusetts General Hospital, Boston, MA 02114, USA; 6 Novartis Institutes for Biomedical Research, Novartis, 4056 Basel, Switzerland; 7 McGovern Institute for Brain Research in the Department of Brain and Cognitive Sciences at MIT, Cambridge, MA 02139, USA

## Abstract

Transcription factor programming of pluripotent stem cells (PSCs) has emerged as an approach to generate human neurons for disease modeling. However, programming schemes produce a variety of cell types, and those neurons that are made often retain an immature phenotype, which limits their utility in modeling neuronal processes, including synaptic transmission. We report that combining *NGN2* programming with SMAD and WNT inhibition generates human patterned induced neurons (hpiNs). Single-cell analyses showed that hpiN cultures contained cells along a developmental continuum, ranging from poorly differentiated neuronal progenitors to well-differentiated, excitatory glutamatergic neurons. The most differentiated neurons could be identified using a *CAMK2A::GFP* reporter gene and exhibited greater functionality, including NMDAR-mediated synaptic transmission. We conclude that utilizing single-cell and reporter gene approaches for selecting successfully programmed cells for study will greatly enhance the utility of hpiNs and other programmed neuronal populations in the modeling of nervous system disorders.

## In Brief

Nehme et al. combine two strong neuralizing factors (transcription factor programming and small molecule patterning) to generate human excitatory neurons from stem cells. They further undertake single-cell and reporter gene approaches to select highly differentiated neurons with increased functionality, augmenting their utility in the modeling of nervous system disorders.

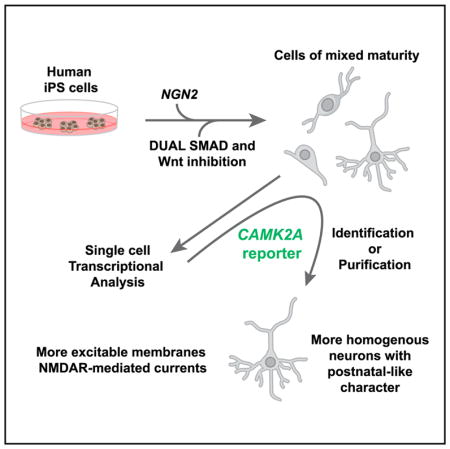

## INTRODUCTION

Progress toward producing more accurate *in vitro* models of human brain cell types continues to be made (Brennand et al., 2015; Paşca et al., 2015). Directed differentiation approaches aim to mimic embryonic development by stepwise specification of neuronal subtypes (Chambers et al., 2009; Espuny-Camacho et al., 2013; Zhang et al., 2013; Ho et al., 2015). In one such strategy, pluripotent stem cells (PSCs) can be neuralized through the inhibition of bone morphogenetic protein (BMP) and transforming growth factor β (TGF-β) signaling (Chambers et al., 2009; Maroof et al., 2013), regionally specified with morphogens, and then allowed to differentiate. While this approach enables cells to transit through cellular states normally observed during embryogenesis, differentiation unfolds slowly. Generation of early post-mitotic forebrain neurons can take as long as 5 weeks, while the production of astrocytes or oligodendrocytes requires even more extended times in culture (Tao and Zhang, 2016).

In contrast, transcription factor-programming approaches rely on ectopic expression of lineage-specific transcription factor(s), in either somatic cells or PSCs, to achieve a rapid cell fate conversion (Son et al., 2011; Mertens et al., 2016). It has been shown that Ascl1, Brn2, and Myt1l can convert mouse fibroblasts into induced neurons (iNs) in as little as 2 weeks (Vierbuchen et al., 2010). More recently, expression of the neuralizing transcription factor NGN2 in human PSCs (hPSCs) was reported to induce an excitatory neuronal identity in a similar time frame (Zhang et al., 2013). While these methods allow more rapid production of human neurons, insight into the heterogeneity of differentiated neurons remains limited. Indeed, using single-cell analysis, it was revealed that, in addition to producing iNs, *ASCL1*-mediated programming also generated muscle cells (Treutlein et al., 2016). Uncertainty surrounding the identity of iN populations raises concerns that programming methods might not produce cells with strong relevance for disease studies.

Cell types made by directed differentiation and programming also share the limitation that they display relatively immature transcriptional phenotypes. For instance, neurons produced by directed differentiation of induced pluripotent stem cells (iPSCs) derived from patients with schizophrenia most resembled human fetal tissue (Brennand et al., 2015). Accordingly, stem cell-derived neurons also displayed immature physiological and synaptic phenotypes. For example, while AMPA receptor (AMPAR)-mediated synaptic transmission was robust in *NGN2*-programmed iNs, only limited expression of N-Methyl-D-aspartate receptors (*NMDARs*), indicative of a more mature state, was detected (Zhang et al., 2013). Increased *NMDAR* expression has routinely been observed only at very late stages of differentiation (up to 145 days in culture) (Gupta et al., 2013; Kirwan et al., 2015). Generation of stem cell-derived neurons with robust NMDAR-mediated synaptic transmission would have specific translational value, as variants in and around the glutamate ionotropic receptor NMDA type subunits 2A and 2B (*GRIN2A* and *GRIN2B*) have been implicated in epilepsy, intellectual disability, autism, and schizophrenia (Schizophrenia Working Group of the Psychiatric Genomics Consortium, 2014; Epi, 2015; Sanders et al., 2015). Improved approaches for generating and identifying neurons with specific and more differentiated phenotypes from PSCs are therefore needed.

Here we tested the utility of combining aspects of directed differentiation and transcription factor reprogramming. We then performed population and single-cell RNA sequencing, as well as electrophysiological recordings, to understand the properties of the resulting cells. Adding extrinsic neuronal patterning to PSCs expressing *NGN2* led to more effective neutralization, resulting in cells that expressed transcription factors expressed in superficial levels of the cortex. Although these cultures were homogenously neuralized, cells existed in transcriptional states that ranged from early progenitor to well-differentiated excitatory neuron states. More differentiated cells expressing *AMPAR* and *NMDAR* subunits also expressed *CAMK2A*, allowing them to be identified using a *CAMK2A::GFP* reporter gene. This approach allowed the isolation of highly differentiated and synaptically active human patterned induced neurons (hpiNs), underscoring the potential utility of this approach for modeling diseases associated with glutamate receptor dysfunction, including schizophrenia, epilepsy, and autism (Yamamoto et al., 2015; Yuan et al., 2015).

## RESULTS

### Patterning of NGN2-Induced hPSCs with Dual SMAD and WNT Inhibition

**Figure 1:**
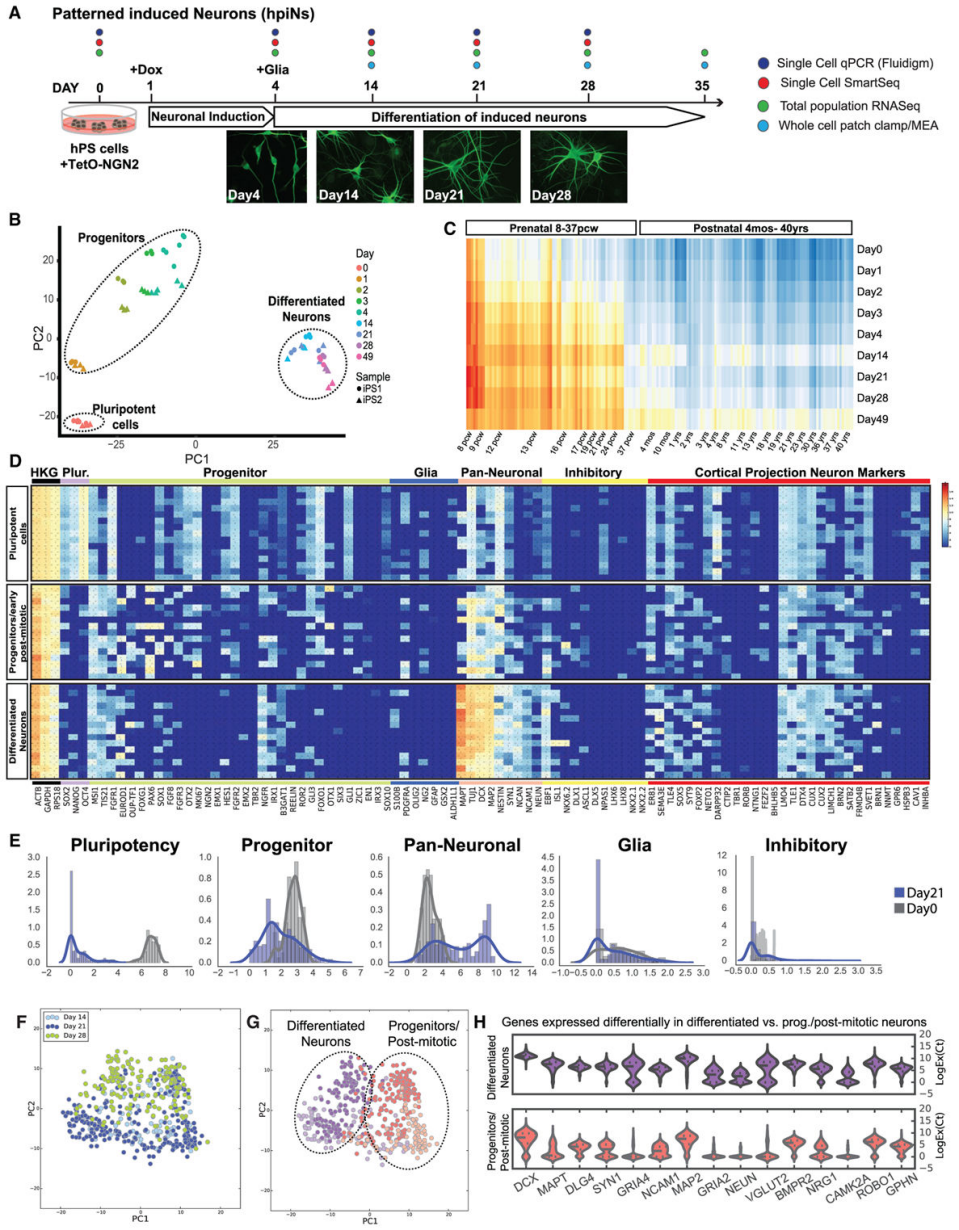
Differentiation over Time in Culture (A) Schematic of hpiN protocol with representative images. NGN2-overexpressing hPSCs are treated with dual SMAD and WNT inhibitors over 3 days, then co-cultured with mouse astrocytes and differentiated into neurons. Scale bar, 10 μm. (B) PCA of population RNA-seq data over 49 days of differentiation. Three main clusters were observed: pluripotent cells, progenitors, and differentiated neurons. (C) Pearson’s correlation of the population RNA-seq of hpiNs over time with cortical structures of the developing human brain. (D) Representative heatmap of single-cell Fluidigm Biomark gene expression data at days 0, 4, 14, and 21 (93 cells at day 0, 51 cells at day 4, 76 cells at day 14, and 239 cells at day 21) (chip1). (E) Density plots. Expression of class-specific genes at day 0 (gray) and day 21 (blue) is shown. (F) PCA of hpiNs harvested at day 14, day 21, or day 28 after induction. (G) Hierarchical clustering (HC) showing that cells from all time points separate into two main clusters, differentiated neurons and progenitor/early post-mitotic cells. (H) Differential gene expression analysis between the more differentiated and less differentiated neurons based on the clusters in (B). *CAMK2A* is one of the differentially expressed genes between the two groups.

Previously, it has been shown that forced expression of the NGN2 transcription factor in hPSCs can induce rapid differentiation into cells with excitable membranes and capable of synaptic function (Zhang et al., 2013). We set out to investigate whether the extrinsic influences of small molecules that inhibit BMP and TGF-β signaling (Chambers et al., 2009; Maroof et al., 2013) could favorably synergize with the activities of NGN2 (Figure 1). To this end, NGN2 expression was induced in TetO-NGN2-T2A-PURO/TetO-GFP lentivirally infected human stem cells by exposure to doxycycline (dox) 1 day after plating. To induce patterning toward a forebrain phenotype, cells were neuralized by inhibiting TGF-β and BMP signaling (treatment with SB431542 and LDN193189), and they were dorsalized by inhibiting Wnt signaling (treatment with XAV939, a tankyrase inhibitor) for 3 days. Puromycin was then applied to select for cells expressing NGN2. The differentiation scheme was performed on both hESC (human embryonic stem cell) and hiPSC lines generated from fibroblasts of healthy individuals (iPS1 and iPS2). At 4 days post-dox induction (day 4), cells were co-cultured with mouse astrocytes to promote neuronal maturation and synaptic connectivity (Pfrieger, 2009; Eroglu and Barres, 2010). Consistent with previous observations (Zhang et al., 2013), changes in cell shape were evident by day 4, with PSCs becoming more polarized and eventually adopting a clear neuronal morphology (Figure 1A).

To examine genome-wide transcriptional changes occurring throughout the differentiation process, we performed population RNA sequencing from two individual hiPSC lines (nine time points, from undifferentiated stem cells until day 49 of induction) (Figure 1A; Table S5). Principal-component analysis (PCA) indicated that, overall, cells clustered along the two principal components according to the extent of time since dox induction, with one cluster comprising days 2–4 and a second cluster encompassing days 14–49. As demonstrated by the tight clustering of biological replicates, these findings were highly reproducible across runs of differentiation and cell lines (Figure 1B). Consistent with NGN2 programming generating predominately excitatory neurons, genes involved in glutamate receptor signaling increased in expression in the differentiated cells over time. This was particularly evident in the case of *AMPAR* subunits (Figure S3C).

Inhibition of SMAD and WNT signaling is known to pattern cultured neurons toward a forebrain identity (Chambers et al., 2009; Maroof et al., 2013). Consistent with previous findings, we failed to detect in the hpiNs expression of genes specific for other lineages, such as spinal motor neurons, hindbrain, MGE/inhibitory neurons, as well as hypothalamic, diencephalic, hippocampal, and dopaminergic markers (Table S1).

To further investigate the effect of small molecule patterning on the iNs, we compared the expression of pluripotency and neuronal progenitor genes by qPCR in day 4 cells that had been subjected to either no small molecule treatment, dual SMAD inhibition (the addition of SB431542 and LDN193189), or dual SMAD and WNT inhibition (the addition of SB431542, LDN193189, and XAV939) in conjunction with NGN2 overexpression. We found that combining dual SMAD and WNT inhibition with NGN2 induction significantly improved several aspects of differentiation relative to NGN2 expression alone. These included downregulation of the pluripotency gene *OCT4*, in addition to significant upregulation of genes expressed in forebrain progenitors, such as *EMX1*, *OTX1*, *OTX2*, *FOXG1*, and *PAX6* (Figure S2E).

We next compared the human BrainSpan dataset, where different human brain areas were transcriptionally profiled over a large developmental window (Brainspan, 2011; Miller et al., 2014), with our population RNA sequencing time course of hpiN differentiation. Pearson correlation between expression data from the brain samples and hpiNs revealed that most profiles of the *in vitro*-derived neurons correlated strongly with the prenatal cortex. Notably, at later stages of *in vitro* differentiation, correlations with human postnatal and even adult stages began to emerge, most prominently in day 49 hpiNs (Figure 1C). Collectively, our initial findings suggest that NGN2 induction synergized well with dual SMAD and WNT inhibition to improve differentiation, resulting in a population of neurons expressing gene products commonly observed in excitatory neurons of the mouse and human cortices, but not factors selectively expressed in other notable brain regions. Furthermore, we found that hpiNs could differentiate sufficiently that at least some molecular features of the postnatal brain appeared to become present.

### Single-Cell qRT-PCR of hpiN Differentiation

To better understand the heterogeneity of differentiating hpiNs, we next turned to single-cell methods for measuring gene expression. Initially, we designed a Fluidigm Biomark Chip for single-cell qRT-PCR with 96 gene-specific probes (Table S2). Each human gene to be measured was selected by orthology with genes previously found to be expressed during discrete developmental epochs of mouse brain development or in select progenitor, glia, or post-mitotic cortical neuronal subtypes (Miller et al., 2014). We rationalized that analyzing the combinatorial expression of these 96 genes in hpiNs would help us to identify the class-specific identity of each differentiating cell (Arlotta et al., 2005; Ip et al., 2010; Saito et al., 2011). To this end, we performed single-cell sorting to harvest individual hpiNs into 96-well plates at several time points (days 0, 4, 14, and 21; Table S5). The transcriptional signature of each cell was then determined using the 96 gene probes described above.

As an initial approach to characterizing the heterogeneity of gene expression among the cells analyzed, we performed PCA, and we found that the first principal component (PC1) accounted for approximately a third of the total variation (27%) in the dataset and could be best explained by the extent of cellular differentiation that had occurred in each cell (Figure S1C). Less differentiated cells clustered together and separately from more differentiated cells (Figure S2A). Subsequent hierarchical clustering of the population structure revealed four clusters that corresponded to three distinct differentiation states (Figures 1D and S1D). The first group of cells expressed genes uniquely active in hPSCs and was composed primarily of day 0 stem cells (n = 100 cells). The second group was composed of cells expressing gene products found in developing brain-progenitor populations, as well as lower abundances of transcripts expressed in many neuronal types. We found that this second cluster was mostly populated by day 4 cells, but also by a subset of cells at day 14 and day 21 of differentiation (n = 216 cells in total). The final group comprised more differentiated cells harvested at later time points (day 14 and day 21, n = 143 cells in total), and it contained a greater abundance of transcripts commonly found in differentiated neurons (Figures 1D, S1C, and S1D). At day 21, the latest time point we initially analyzed, we found that 45% of hpiNs fell within the second cluster of apparent progenitors, while 55% of cells appeared to have become more differentiated neuronal cells (third cluster).

We next compared the expression of specific groups of genes between cells harvested at day 0 and day 21. Density plots showed a decrease of pluripotency and progenitor genes in day 21 hpiNs relative to day 0 cells (Figures 1D and 1E). Conversely, pan-neuronal gene expression was increased (Figure 1E; Table S3). In agreement with our population RNA sequencing data, transcripts expressed in mouse upper layer cortical projection neurons were increased in their abundance, while transcripts found in deep layer mouse cortical neurons were not enriched in day 21 hpiNs (Figures 1E and S1F). Strikingly, *DCX*, *MAP2*, and *NEUN* expression significantly increased up through day 14, indicating that significant neuronal differentiation was already occurring between day 4 and day 14 (Figure S1F). Furthermore, hpiNs did not express significant levels of inhibitory neuron markers or glial markers at any time point during differentiation (Figures 1D and 1E). These results were reproducible across an independent cell line (iPS2) (Figures S2A and S2B; n = 660 cells). Finally, to validate the single-cell Fluidigm results at the protein level, we examined the expression of a subset of these gene products by immunostaining. We confirmed the expression of transcription factors expressed in upper layer cortical projection neurons of the mouse, such as CUX1, CUX2, and BRN2, but not markers expressed in the deep layers, such as CTIP2 (Figures S1A and S1B). Taken together, these results suggested that the iNs were homogeneously patterned but were heterogenous with respect to the extent of their differentiation.

### Comparison between Transcriptional Profiles of Progenitors and Differentiated Neurons

When we examined individual genes, we noted that a subset of progenitor markers remained expressed in day 21 hpiNs (Figures 1D, 1E, and S1F). Intrigued by the persistence of progenitor features as well as the emergence of mature features in the more differentiated neuronal population, we designed a second Fluidigm Biomark Chip (chip 2) with genes associated with cortical glutamatergic neurons, as well as neurochemical and synaptic markers (Table S2). We then profiled single neurons differentiated for 14, 21, and 28 days (511 cells analyzed in total; Table S5). PCA and hierarchical clustering of gene expression data confirmed that differentiation state was the main driver segregating the clusters (Figures 1F and 1G). In particular, cells segregated into two main clusters, which were classified as progenitor/early post-mitotic and differentiated based on the PC loadings. However, each cluster contained cells from each of the time points, suggesting that some progenitors did not transition toward a more differentiated state over time.

We next assessed the reproducibility of the hpiN differentiation scheme among three human iPSC lines (iPS1, iPS2, and iPS3) and one hECS line (ES1), as well as across biological replicates (n = 891 cells examined; Figure S2; Table S2), and we found highly similar patterning (Figures S2C and S2D). Furthermore, PCA indicated that, regardless of whether small molecule patterning was added to NGN2 programming or the length of time that dox induction was applied, a population of neuronal progenitors that seemed resistant to further differentiation remained present (Figures S2F–S2H).

Using our Fluidigm datasets, we next performed differential gene expression analysis to identify candidate marker genes for the most molecularly differentiated neurons. The top differentially expressed genes between progenitors and more differentiated neurons were known pan-neuronal markers, such as *MAP2*, *MAPT*, and *NEUN*, or synaptic markers, including *SYN1*, *DLG4*, *GPHN*, and *CAMK2A* (Figure 1H). Upon closer examination, we found that *GRIA1*, *GRIA4*, *NEUN*, and *CAMK2A* expression appeared to be present in the differentiated cluster but nearly or completely absent in the progenitor cluster. Of these, NEUN is a commonly used neuronal marker, but, given that it is a nuclear protein, it did not meet our criteria as a marker with potential for isolation of differentiated neurons. After canvassing the literature for any known role in postnatal mouse brain and mature synaptic function for the candidates on our gene list, *CAMK2A* stood out because of its well-known expression at postnatal stages in mouse (Lein et al., 2007). Camk2a-GFP mice have also been used to identify postnatal, post-mitotic glutamatergic neurons in rodents (Lein et al., 2007; Wang et al., 2013), as well as mature forebrain neurons (Shcheglovitov et al., 2013). Encouraged by these connections, we queried *CAMK2A* expression during human brain development in the BrainSpan dataset (Miller et al., 2014), and we found that its expression increased markedly after birth.

### Single-Cell Molecular Analysis of CAMK2A-Expressing hpiNs

**Figure 2:**
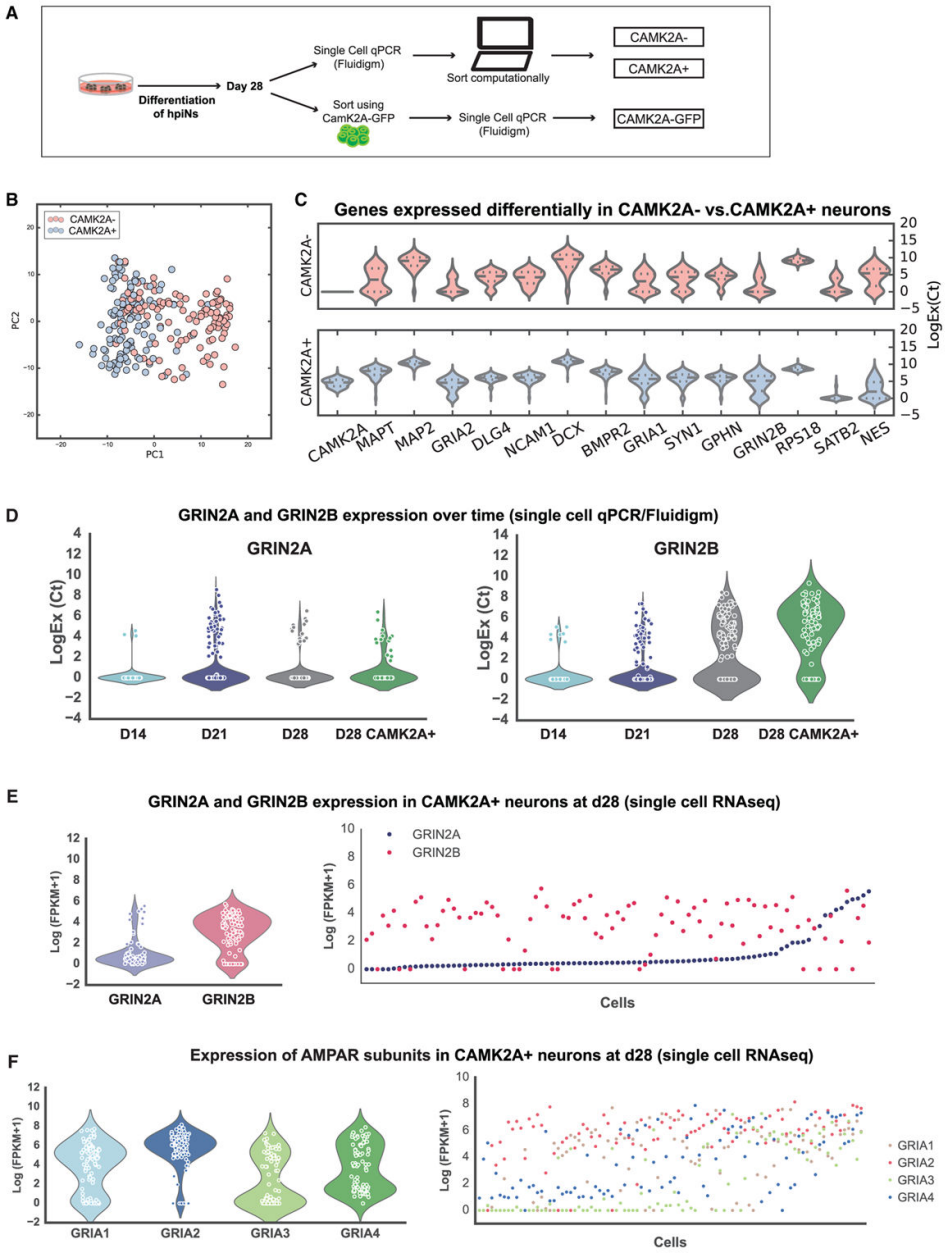
*CAMK2A* as a Marker of the More Differentiated Population (A) Schematic describing the isolation of *CAMK2A+* cells, either computationally or with a GFP reporter. (B) PCA of *CAMK2A+* and *CAMK2A*− cells harvested at day 28. (C) Violin plots of genes differentially expressed in *CAMK2A+* and *CAMK2A*− cells. (D and E) Violin plots and scatterplots showing the expression of GRIN2A and GRIN2B over time in hpiNs and *CAMK2A+* neurons at day 28, both detected by single-cell Fluidigm Biomark (D) and by single-cell RNA-seq (E). (F) Violin plots and scatterplots showing the expression of the *AMPAR* subunits in single cells at day 28.

To investigate whether *CAMK2A* expression could serve as a useful marker for the more differentiated hpiNs, we transduced the cells with a lentivirus expressing GFP under the control of the *CAMK2A* promoter. We then assayed the expression of the Fluidigm chip 2 probe set (Table S2) in day 28 hpiNs. Single cells were either sorted for *CAMK2A::GFP* expression (81 cells) or *TetO-GFP* (161 cells); the *TetO-GFP* cells were then computationally sorted for the expression of *CAMK2A* (242 cells analyzed in total; Figure 2A; Table S5). RNA sequencing at day 28 post-dox induction revealed that 52% of hpiNs expressed endogenous *CAMK2A* (fragments per kilobase of transcript per million mapped reads [FPKM] > 0), while 20%–30% of the counted cells expressed *CAMK2A-GFP*. Nearly all *CAMK2A::GFP*-sorted cells expressed endogenous *CAMK2A*, validating that the *CAMK2A::GFP* construct was accurately reporting on endogenous *CAMK2A* expression (92%) (Figure S3D). When we compared the molecular profile of these three categories of cells, PCA and differential gene expression analysis showed that the *CAMK2A*+ cells expressed higher levels of pan-neuronal and synaptic genes compared to the *CAMK2A*− cells. This was true both for cells sorted computationally based on their endogenous expression of *CAMK2A* (*CAMK2A*+) and cells isolated by flow cytometry using the *CAMK2A::GFP* reporter (Figures 2B and 2C).

We next carried out single-cell RNA sequencing on the same populations following the Smart-seq2 protocol (Picelli et al., 2013, 2014). Because of the implication of NMDAR in various disorders of the nervous system, we examined the expression of NMDAR subunits *GRIN2A* and *GRIN2B*, as well as the AMPAR subunits *GRIA1*, *2*, *3*, and *4* in the iNs by single-cell qPCR (n = 242 cells) as well as single-cell RNA sequencing (RNA-seq) (n = 93 cells) (Figures 2D–2F). We found that *GRIN2B* was robustly expressed in most *CAMK2A*+ cells at day 28 (83% of the cells, with an FPKM > 1). Conversely, *GRIN2A* was expressed in most cells at relatively low levels (95% of cells, with an FPKM > 0), and it was expressed in only a subset of the cells at higher levels (22% of the cells, with an FPKM > 1) (Figures 2D and 2E). We then compared these expression profiles to their developmental expression in the human cerebral cortex (http://www.brainspan.org; Miller et al., 2014) (Figure S3A). Consistent with previous reports (Traynelis et al., 2010), we found that *GRIN2B* expression was detected in the human cerebral cortex in prenatal stages, with some persisting expression into the postnatal stages. *GRIN2A*, on the other hand, was detected almost exclusively in postnatal stages.

Moreover, by comparing hpiNs with hpiNs produced without dual SMAD and WNT inhibition, we found that, in both hiPSC lines examined, the expression of the NMDAR subunit *GRIN2B* was significantly higher in neurons generated in the presence of patterning small molecules (Figure S2I). This suggests that patterning combined with NGN2 induction can synergize to promote expression of NMDAR subunits.

**Figure 3:**
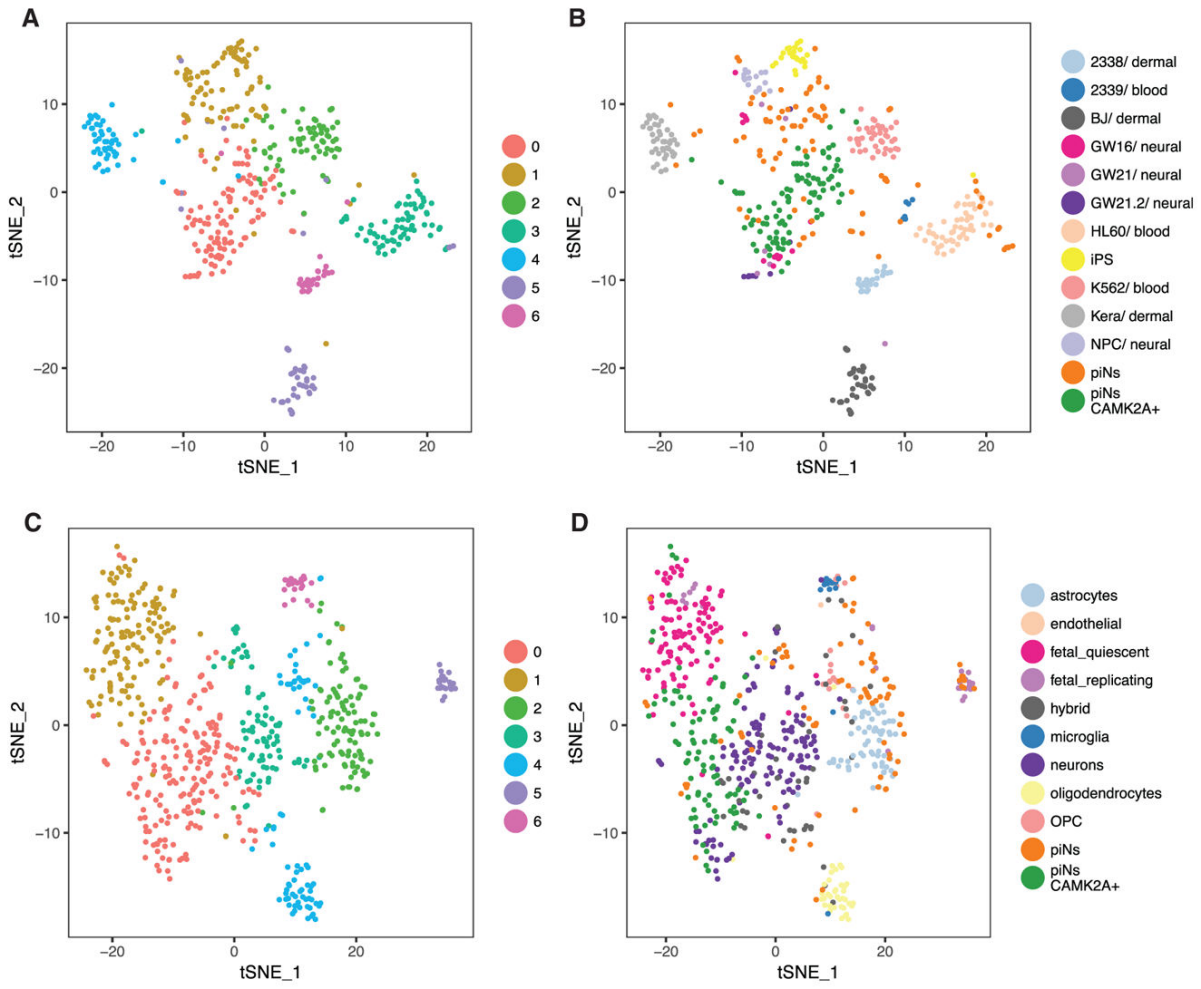
CAMK2A+ hpiNs Resemble Fetal and Adult Neurons (A and B) Comparison to Pollen et al., 2014 dataset. The majority of CAMK2A+ hpiNs cluster with fetal neurons (GW16-21) in the reference dataset. (A) tSNE labeled by graph-like clusters and (B) tSNE labeled by cell types. (C and D) Comparison to Darmanis et al., 2015 dataset. Unsorted cells contribute to neuronal and non-neuronal clusters while CAMK2A+ hpiNs cluster with fetal and adult neurons in the reference dataset. (C) tSNE labeled by graph-like clusters and (D) tSNE labeled by cell types.

To gain a deeper understanding of how similar hpiNs are to *in vivo* cell types, we then compared their expression profiles to a publicly available dataset of single-cell RNA-seq of the developing cortex (Pollen et al., 2014). We found that, while unsorted hpiNs contributed to both neuronal and non-neuronal clusters (such as neural progenitor cells [NPCs]), *CAMK2A*+ hpiNs most closely resembled neurons (Figures 3A and 3B). Next, we compared hpiN transcriptional profiles to a second reference dataset, which included RNA-seq of several cell types isolated from adults, in addition to embryonic cell types (Darmanis et al., 2015). Consistent with our previous results, we found that unsorted hpiNs clustered closely with neurons and other cell types present in the brain. However, *CAMK2A*+ hpiNs clustered with both fetal and adult neurons in the reference dataset (Figures 3C and 3D). These analyses validated the utility of the *CAMK2A::GFP* reporter in demarcating the neuronal population, and the identification of more differentiated neurons.

### Network Activity of hpiNs as a Function of Neuronal Differentiation

**Figure 4:**
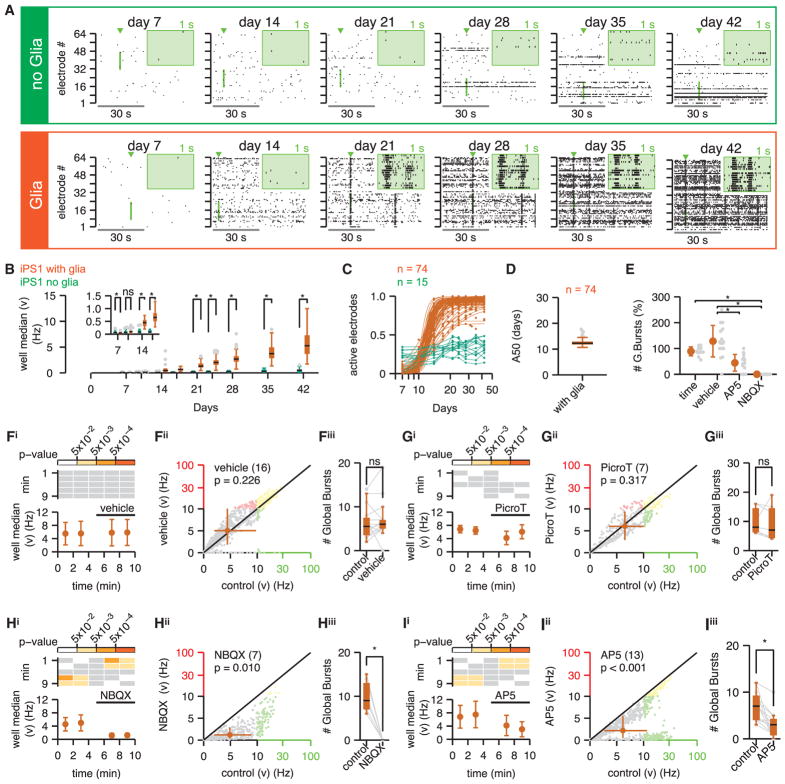
hpiNs Form Active Networks that Are Glutamate Receptor Dependent Neuronal activity was routinely sampled on MEA plates for 42 days. (A) Representative raster plots of spontaneous activity. Neurons were cultured in isolation (top, green) or with glia co-culture (bottom, orange). Top right insets (green background) show spikes detected on 16 electrodes with an expanded 1-s time base (the exploded region is both highlighted in and marked by a green arrowhead above the main figure). (B–E) Network activity of hpiNs. (B) Spike count (mean number of spikes in a 10-s period). The activity of neurons derived in isolation (n = 15 cultures from 3 differentiations) is compared to those co-cultured with glia (n = 74 cultures from 6 differentiations). (C) Proportion of electrodes detecting spontaneous activity, against the number of days post-induction. For neurons cultured with glia, the data were well fit by a sigmoid function. (D) Summary of the A50 from the sigmoid curve fits. The neurons cultured in the absence of glia have not been curve fit. (E) Number of burst parameters across conditions; time, time base control group that did not receive vehicle or drug (n = 12 cultures from one differentiation). (F–I) Contribution of ionotropic glutamate and GABA receptors to spontaneous activity (at day 42) for neurons cultured with glia. Electrode mean spike count (/10 s) recorded over 10 min is shown. The solid bar above the plots highlights the addition of vehicle (F, dd.H20, n = 16 cultures from 3 differentiations) or drug (G, picrotoxin, n = 7 cultures from 2 differentiations; H, NBQX, n = 7 cultures from 2 differentiations; I, AP5, n = 13 cultures from 3 differentiations). (F–Ii) Top significant p values are shown (p values > 0.05 are in white, gray indicates that no statistical comparisons have been made). (F–Iii) Mean electrode spike counts per electrode recorded during the baseline control period (fourth minute) are plotted against the spike count recorded during the test period (ninth minute). The diagonal line of equality is plotted in black, and the orange filled circle (and bars) marks the median (and Q1–Q3). Plots are split into 4 quadrants; for paired spike counts ≤100 (gray data), the ordinate and abscissa are both plot on a linear scale, and when paired spike counts are both above 100 they are plot on log-log scale (yellow data). Otherwise data are plot on a mixed linear-log/log-linear scale (red and green, respectively). (F–Iiii) Effect of vehicle/antagonist application on the number of global bursts is shown. Bursts are analyzed for 2 min at the end of the baseline period and for 2 min after drug/vehicle application. Raw data are plotted in gray; *p value ≤ 0.05; ns, not significant.

We next set out to comprehensively investigate the functional properties of hpiNs. We first used multielectrode arrays (MEAs) to monitor differentiation of stem cells into functional neurons at a population level and over time. To this end, we seeded hPSCs on 8 × 8 MEA grids, each with 64 microelectrodes, in the absence and presence of mouse glia (Table S5). The example raster plots, which provide a graphical representation of extracellular action potential trains, showed sparse spiking as early as day 7 (Figure 4A). The spike rate then continued to increase throughout the 42-day recording period (Figure S4A). Notably, co-culturing hpiNs with glia led to significantly higher spiking rates (day 10 onward; Figures 4A, 4B, S4A, and S4B) and the development of pronounced MEA-wide/global bursts that were indicative of neuronal connectivity, between days 14 and 21 (Figure 4A). Overall, we found that half of the electrodes were functional by 12.5 when cultured with hpiNs and glia (Figures 4D and S4D). Two additional cell lines (iPS2 and ES1) characterized in this study both matured at a comparable rate, illustrating the reproducibility of activity achieved with this method (Figures S4C and S4D).

### A Physiologically Functional Synaptic Network

Previous studies have suggested that much of the activity found in neurons produced with NGN2 expression is mediated through AMPARs (Zhang et al., 2013). Consistent with this notion, we observed that application of the AMPAR competitive antagonist NBQX (2,3-dihydroxy-6-nitro-7-sulfamoyl-benzo[f]quinoxaline-2,3-dione) attenuated spontaneous spiking rates and blocked the occurrence of network-wide bursts (Figures 4H and S4E) compared to the vehicle (Figures 4F, S4E, and S4F). A complete blockade of the residual activity by tetrodotoxin (Figures S4H and S4I), a voltage-gated sodium channel blocker, confirmed that these spikes were extracellular action potentials dependent on the activity of sodium-gated calcium channels. The AMPAR-independent spikes were likely to be occurring due to spontaneous activity in a few residual neurons.

We next wondered whether network effects could also be NMDAR mediated. Indeed, application of the NMDAR-selective competitive antagonist D-AP5 significantly reduced spike rate and reduced the occurrence of network-wide bursts (Figures 4E, 4I, and S4Eiv). Reduction of the spike rate by an NMDA antagonist is direct evidence for surface expression of functional NMDARs in the hpiNs and their contribution to the generation of spontaneous neuronal activity. Notably, while NMDAR antagonism reduced the spike rate and burst activity of the neurons, it had no significant effect on the number of spikes within the network bursts (data not shown). In contrast, application of GABA_A_ receptor antagonist picrotoxin did not significantly affect firing rates (Figures 4G and S4Eii) or the occurrence of network-wide bursts. Thus, GABA_A_ receptor-mediated synaptic transmission did not contribute significantly to the synaptic transmission in the iNs. Taken together, these results confirmed our conclusions from transcriptional profiling, and they demonstrated that both AMPARs and NMDARs mediate excitatory synaptic transmission in hpiNs.

### CAMK2A Expression Demarcates hpiNs with More Mature Electrophysiological Properties

**Figure 5:**
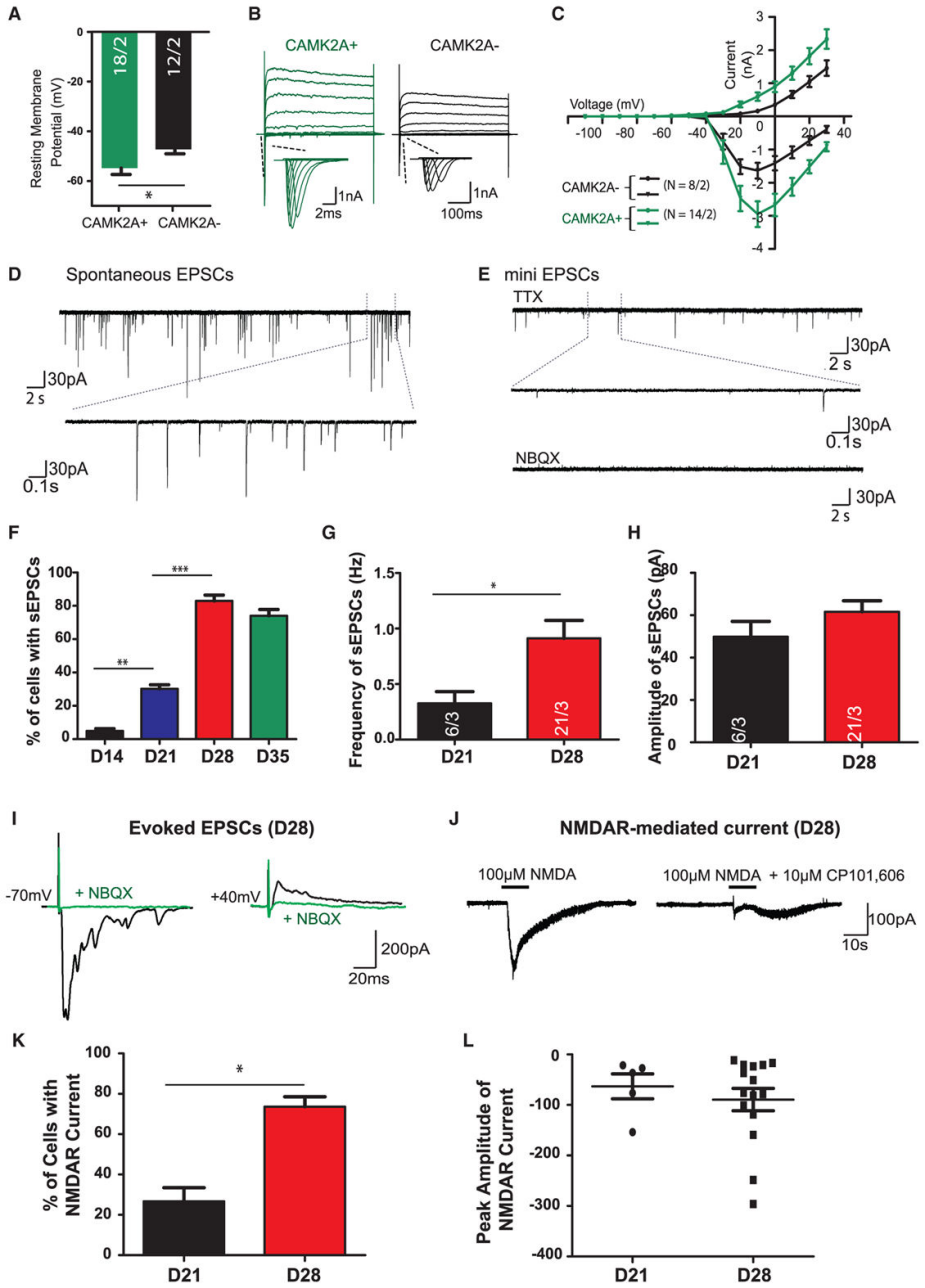
Time Course Characterization of the Electrophysiological Membrane Properties of *CAMK2A+* Neurons over Time (A) Comparison of electrophysiological membrane properties of *CAMK2A::GFP+* and *CAMK2A::GFP*− at day 21. (B) Representative traces of whole-cell voltage-clamp Na^+^ and K+ currents recorded in a *GFP+* cell and a GFP− at day 21. Cells were subjected to a 10-mV step depolarizations from −110 to +30 mV at a −70-mV holding membrane potential. (C) Quantification of current/voltage (I/V) curves of Na^+^ and K+ currents. (D and E) Representative traces of (D) spontaneous EPSCs and (E) miniature EPSCs in the presence of 1 μM TTX at day 28. Lower panel illustrates expansions of selected events. (F) Percentage of cells exhibiting sEPSCs is significantly increased over time from day 14 to day 28. (G) Quantification of the frequency of sEPSCs shows a significant increase at day 28 as compared to day 21. (H) Quantification of the peak amplitude of sEPSCs recorded at day 21 and day 28. (I–L) *CAMK2A+* neurons mainly express GRIN2B-containing NMDA receptors. (I) Block of spontaneous EPSCs by 10 μM NBQX at day 28. Representative traces show evoked EPSCs measured at a holding membrane potential of −70 mV (left panel) followed by a holding potential of +40 mV at day 28; the evoked EPSCs at both holding membrane potentials were blocked by 10 μM NBQX. (J) Representative traces of whole-cell currents elicited by the application of a saturating concentration of NMDA (100 μM). Application duration is indicated by solid bars above traces. (K) Summary of the percentage of neurons that exhibit NMDA receptor-mediated currents at day 21 and day 28. (L) Summary of the NMDAR-mediated current density (pA/pF) at day 21 and day 28. Data are means ± SEMs. *p < 0.01, **p < 0.005, and ***p < 0.001, unpaired Student’s t test.

To investigate whether the *CAMK2A* reporter gene could be used to identify more differentiated cells with additional functionalities, we subjected cells with or without expression of *CAMK2A::GFP* to whole-cell patch clamp at different time points (days 14, 21, and 28; Table S5). Indeed, electrophysiological characterization of *GFP*+ neurons showed more mature membrane properties as compared to neighboring *GFP*− cells. *GFP*+ neurons showed significantly more negative resting membrane potential (RMP), compared to *GFP*− cells, and also significantly larger membrane capacitance (Figures 5A, S5A, and S5B). We next quantified action potential (AP) firing properties by giving step current injection in current-clamp mode to elicit APs. We found that *GFP*+ cells at day 21 post-differentiation showed significantly more hyperpolarized AP threshold and a trend toward increased average spike numbers per pulse, suggesting more mature AP-firing properties (Figures 5B and S5C). No significant changes were observed in the AP peak amplitude and AP half-width, most likely due to the intrinsic variability of the two AP parameters (Figure S5D). *CAMK2A*+ neurons reached a steady level of maturation at day 28 when APs exhibited mature and homogeneous firing patterns.

We further compared voltage-gated sodium (Na^+^v) channel and potassium (K^+^v) channel currents between *GFP*+ cells and *GFP*− cells. Consistent with the more mature AP-firing properties, *CAMK2A*+ cells showed larger voltage-dependent Na^+^ and K^+^ currents than those in *GFP*− cells (Figure 5C).

### Membrane Properties and Synaptic Transmission in hpiNs

We next performed whole-cell patch-clamp recording at days 14, 21, 28, and 35. The resting membrane potential of *GFP*+ neurons became increasingly negative over time (Figure S5E), reaching statistical significance between days 21 and 28. Moreover, when neurons were subjected to 10-mV step depolarization, we detected large peaks of the fast inward Na^+^ current (confirmed by Na^+^v blocker 1 μM tetrodotoxin [TTX]), followed by long-lasting outward K+ currents (confirmed by K^+^v blocker 10 μM tetraethylammonium chloride [TEA]) at day 28. We further quantified AP-firing properties by giving step current injection of 20 pA in current-clamp mode to elicit APs. At the first 3 weeks after differentiation, neurons were not able to produce multiple APs in response to a prolonged depolarizing current step (Figure S5F). However, by day 28 nearly 75% of the cells recorded were capable of producing multiple APs or AP trains. Analysis of AP-firing properties consistently showed time-dependent maturation, comparable to the temporal expression of Na^+^v and K^+^v (Figure S5G). Thus, the hpiNs exhibited a steady ongoing process of membrane electrical property maturation, in which a pronounced shift in a range of electrophysiological parameters occurred between day 21 and day 28 post-differentiation. A reproducible electrical maturation pattern was observed across hPSC lines and across biological replicates (Table S4). Overall, the parameters of AP quantification along with RMP of hpiNs at day 28 to day 35 post-differentiation were comparable to rodent cortical neurons at an early postnatal development stage (Spitzer, 1994; Oswald and Reyes, 2008).

### Functionality of AMPAR and NMDAR in hpiNs

We next asked whether the synaptic properties of hpiNs reflected the complement of synaptic components whose transcription we had detected by RNA-seq. We investigated basal synaptic transmission; AMPAR mediated spontaneous excitatory postsynaptic currents (sEPSCs) that were AP dependent, and AMPAR mediated miniature EPSCs (mEPSCs) in the presence of TTX to block APs. Approximately 5% of cells displayed sEPSCs at day 14. We detected a dramatic increase in the percentage of cells showing sEPSCs (85%) at day 28, suggesting that the synaptic transmission level peaked between day 14 and day 28 (Figure 5F). Concordantly, the frequency and amplitude of sEPSCs were higher at day 28 (Figures 5G and 5H). These spontaneous synaptic activities were blocked by AMPAR antagonist NBQX, as well as by TTX (Figures 5D and 5E).

### NMDA Receptor-Mediated Currents in CAMK2A+ hpiNs

We next went on to perform pharmacological characterization of NMDA receptor-mediated currents in *CAMK2A::GFP+* neurons at days 14, 21, 28, and 35. As shown in Figure 5I, the evoked EPSCs were blocked by NBQX at both −70- and +40-mV holding potentials. This suggests that the synaptically evoked EPSCs are mainly contributed by the activation of AMPAR type, but not NMDA-type glutamate receptors, at day 28. However, when we implemented fast, exogenous application of 100 μM NMDA to the hpiNs by pressure injection with a Picospritzer, we found that approximately 20% of the cells recorded displayed NMDAR-mediated currents at day 21 (Figure 5J). NMDA receptors are found in both synaptic and extra-synaptic locations on neurons, and the best-characterized extra-synaptic NMDA receptors include GRIN1 and GRIN2B subunits. The saturating concentration of NMDA-induced current responses was largely blocked by 10 μM CP101, 606, a GRIN2B subunit-selective NMDA receptor antagonist (Figure 5J). We again observed a large shift in electrophysiological properties between day 21 and day 28 neurons: about 70% of cells exhibited NMDAR-mediated current upon NMDA application at day 28 (Figure 5K). As shown in Figure 5L, the current density of NMDAR-mediated current was significantly enhanced as compared to day 21, suggesting that more functional GRIN2B-containing NMDARs were expressed in day 21 neurons (in agreement with the high expression level of *GRIN2B* at day 21 and day 28, with a subset of cells displaying *GRIN2A* expression at day 28; Figure 2). Strikingly, these results suggest that *CAMK2A* expression can be used as a tool to isolate a more mature subset of hpiNs capable of NMDAR-mediated synaptic transmission. Furthermore, our findings suggest that, while a subset of *CAMK2A*− progenitor cells may not further differentiate, the physiological properties of *CAMK2A*+ cells do continue to mature for some time.

### Expression of Disease-Associated Synaptic Genes

**Figure 6:**
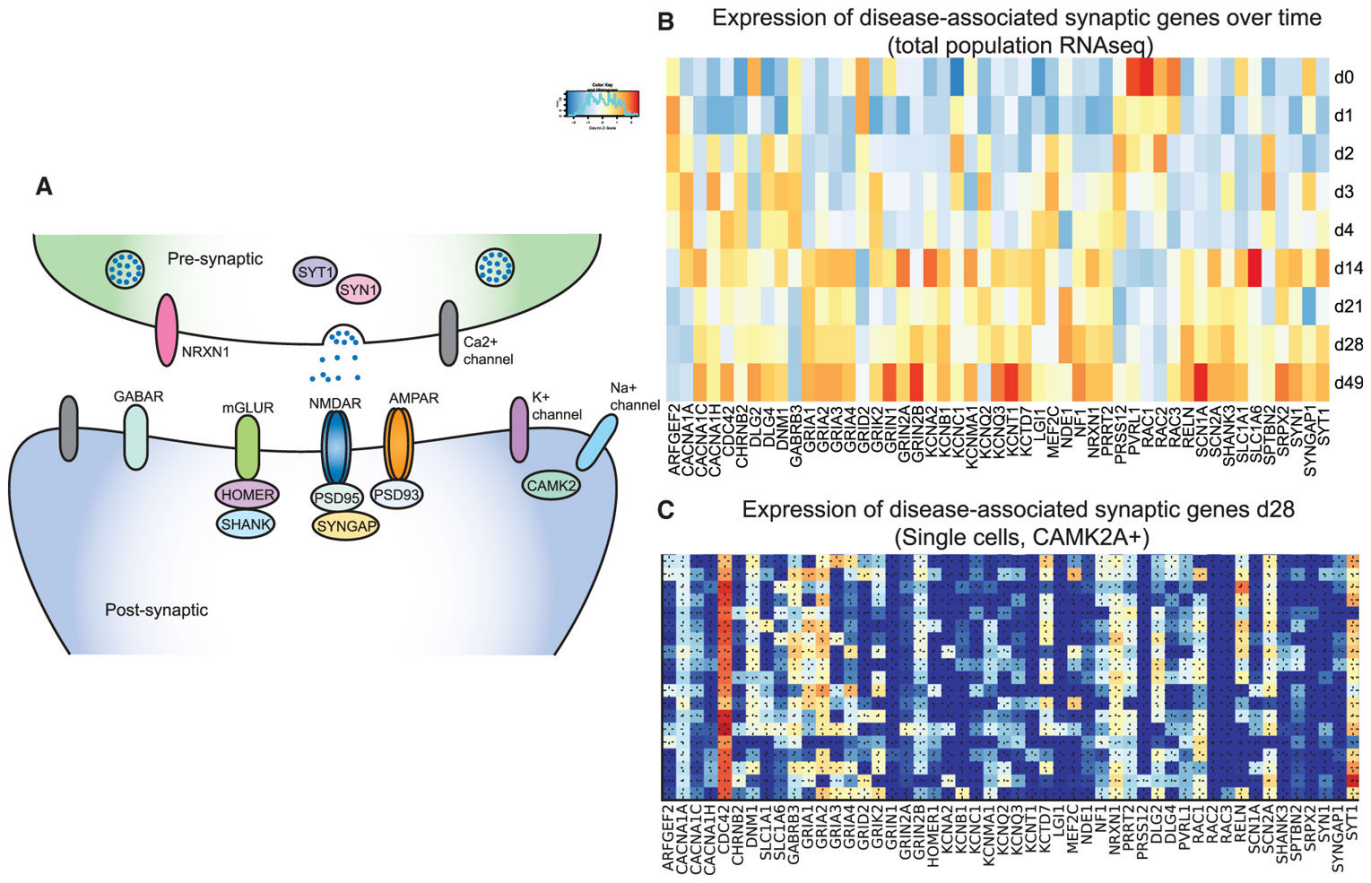
Expression of Disease-Associated Synaptic Genes in the Derived Neurons (A) Schematic of a synapse highlighting the synaptic components of interest. (B) Expression of the disease-associated genes over time based on population RNA-seq data. (C) Expression of the disease-associated genes in *CAMK2A+* single cells (Smart-seq2 data) at day 28.

Large-scale sequencing studies have shown that mutations in genes whose products function in excitatory synapses are enriched in patients with neurodevelopmental diseases (Figure 6A). For example, as evidenced by exome sequencing studies, mutations in NMDARs are enriched in patients with forms of epileptic encephalopathies (Epi, 2015). Similarly, a broader set of synaptic genes has been proposed to play a role in autism, again through exome sequencing studies (Sanders et al., 2015). Association studies have also implicated some of these factors in aspects of schizophrenia and intellectual disability (ID) (Schizophrenia Working Group of the Psychiatric Genomics Consortium, 2014). To explore the potential of our hpiN-profiling dataset as a resource for disease-modeling studies, we determined the expression of disease-associated genes by total population RNA-seq and in single sorted day 28 CAMK2A-expressing hpiNs (Figures 6B and 6C). We found that many disease-relevant genes were expressed in the hpiNs and that the expression of numerous genes (including AMPAR subunits *GRIA1*, *2*, and *3* and NMDAR subunits *GRIN1* and *GRIN2B*) increased over differentiation time.

## DISCUSSION

We describe here a rapid and reproducible differentiation scheme that combines aspects of NGN2 programming in hPSCs (Zhang et al., 2013) with developmental patterning through the inhibition of SMAD and WNT signaling (Chambers et al., 2009; Maroof et al., 2013). This approach generated a homogenously patterned population of cells differentiating toward a cortical excitatory identity. Their properties included robust expression of *AMPARs* and *NMDARs*, as well as mature synaptic function. We also provide an extensive minable dataset comprising single-cell and total population RNA-seq libraries. This comprehensive resource can be used to determine whether specific genes/disorders can be studied in hpiNs and which time point(s) would be most suitable for such studies.

We find that hpiNs express many of the genes associated with an upper layer cortical projection neuron identity in mouse and human brains (Lake et al., 2016; Tasic et al., 2016). Comparing our population and single-cell transcriptomes with stages of human brain development, we find that the hpiN population overall is the most strongly correlated with the prenatal brain, with emerging postnatal and adult features in the *CAMK2A*-expressing hpiNs. These characteristics make hpiNs an attractive model system for modeling aspects of disorders such as autism or schizophrenia, where layer 3 pyramidal neurons in patients show reduced spine density (Lewis et al., 2003). Consistent with previous reports that glial cells promote neuronal maturation and synaptic activity (Pfrieger, 2009; Eroglu and Barres, 2010; Tang et al., 2013), we found that co-culturing the stem cell-derived neurons with mouse glia resulted in an increase in firing, as well as in synchronous activity that was reminiscent of bursting activity. This increased activity coincided with an increase in expression of genes that are involved in neuronal activity and synaptic connectivity.

Heterogeneity of stem cell-derived cell types plagues most if not all differentiation or programming approaches reported to date, and it is a major impediment in their use for disease-related studies. Single-cell RNA-seq technologies are now providing important insight into the extent of this heterogeneity. Yao et al. (2017) performed clonal analysis in differentiating hESCs to map a lineage tree of *in vitro* brain development. Comparing clustered single-cell transcriptomes with mouse and human gene expression data, they found that a wide-ranging set of progenitor cells, as well as midbrain/hindbrain and cortical subtypes, was present in such cultures (Yao et al., 2017). For direct reprogramming using the pioneer factor ASCL1, Treutlein et al., (2016) found that transgene silencing prevented a subset of fibroblasts from becoming reprogrammed and that a second population of fibroblasts unexpectedly activated a myocyte-like transcriptional program. These findings illustrate the strength of single-cell technologies to provide insight into the molecular heterogeneity of stem cell-derived populations, as well as the challenges associated with harnessing their translational utility.

We found that, although the hpiNs were homogeneously patterned, indicative of a similar neuronal identity, they were heterogeneous with respect to their extent of differentiation. Notably, we detected the expression of progenitor genes prior to neuronal genes, suggesting the presence of a progenitor population. We found that progenitor cells persisted at later time points, indicating that extending time in culture may be of limited use for eliminating them. The reason behind this heterogeneity is unclear but could be due to differences in NGN2 expression between cells. By designing a single-cell profiling approach aimed to resolve progenitor-like cells from more differentiated hpiNs, we were able to resolve much of this heterogeneity. We further identified *CAMK2A* expression as a marker for the more differentiated hpiNs, which we also found had more functional synaptic properties. This approach could be generalizable to resolve progenitor subsets from more differentiated derivatives with superior functional properties in other differentiation or direct reprogramming protocols. Most importantly, our molecular and functional analyses revealed robust expression of NMDAR subunits and NMDAR-mediated synaptic properties in day 28 hpiNs. NMDAR subunits have been implicated in many neurodevelopmental and psychiatric disorders through exome sequencing and association studies (Schizophrenia Working Group of the Psychiatric Genomics Consortium, 2014; Epi, 2015). Being able to rapidly generate homogeneous populations of cortical neurons that robustly express these receptors will greatly facilitate the study of brain disorders.

## EXPERIMENTAL PROCEDURES

### Differentiation of hpiNs

hPSCs were co-infected with TetO-Ngn2-Puro and TetO-GFP (gift from the Wernig Lab) and reverse tetracycline-controlled transactivator (rtTA), and were plated at a density of 100,000 cells/cm^2^ with rock inhibitor Y27632 (Stemgent 04-0012). Day 1 cells were differentiated in KSR media with 10 μM SB431542 (1614, Tocris), 2 μM XAV939 (04-00046, Stemgent) and 100 nM LDN-193189 (04-0074, Stemgent) along with doxycycline hyclate (2 μg/mL) (maintained for the entire differentiation process, unless noted). Day 2 media were 50% KSR+SB/XAV/LDN and 50% N2 supplemented with puromycin (5 μg/μL), and differentiation media were as previously described (Maroof et al., 2013). Day 3, N2 media, day 4, neurobasal media (Gibco) supplemented with B27 (50×, Gibco), brain-derived neurotrophic factor (BDNF), ciliary neurotrophic factor (CTNF), and glial cell-derived neurotrophic factor (GDNF) (R&D Systems 248-BD/CF, 257-NT/CF, and 212-GD/CF at 10 ng/mL). When co-cultured with glia, day 4 cells were dissociated with accutase and plated at a density of 40,000/cm^2^ with mouse primary cortical glial cells (seeded at 70,000/cm^2^ glia) on geltrex-coated plates. Primary glial preparations from post-natal day (P)0–P2 mouse pups were obtained as previously described (Di Giorgio et al., 2008).

### Single-Cell Expression Profiling and Transcriptomics

Cells were dissociated using accutase supplemented with DNase (Worthington Biochemical, LK003170). A 100-μm nozzle was used for sorting. Cells were sorted by BD Facs Aria, 20 psi, 40–100 ev/s into 96-well plates (one cell per well) containing 5 μL lysis buffer. For Fluidigm Biomark, cells were single-cell sorted into 5 μL of 10 mM Tris (pH 8.0), 0.1 mM EDTA supplemented with SUPERase•In (0.1 U/μL final, Ambion, AM2696), and 0.5% NP40 (Thermo Scientific, PI-28324). For Smart-seq2 library prep, cells were single-cell sorted into 5 μL 1×TCL buffer (QIAGEN, 1031576). After sorting, plates were immediately sealed, spun down for 5 min at 550 × *g*, and flash frozen on dry ice. Sorted cells were then stored at −80°C. Details on single-cell expression profiling and transcriptomics analysis are provided in the Supplemental Experimental Procedures. To compare the piN cells to publicly available single-cell reference datasets (Pollen et al., 2014, Darmanis et al., 2015), we performed integrated analysis using Seurat version (v.)2.1

### Statistics

#### qPCR

Multiple comparison ordinary one-way ANOVA was performed with Prism 6 (GraphPad, La Jolla, CA, USA).

#### Single-Cell Expression

Analysis of data and visualization of results were performed using Python packages (Numpy 1.7.1, Pandas 0.12.0, SciPy 0.12.0, and Matplotlib 1.2.1) and R statistical software (3.2.3).

#### MEA

All descriptive statistics and statistical tests were performed in MATLAB environment (v.8.3, R2014a, MathWorks), using the Statistics Toolbox (v.9.0, R2014a, MathWorks). Additional details are provided in the Supplemental Experimental Procedures.

#### Institutional Permissions

iPS1 and iPS2 were generated from fibroblasts collected in Umea, Sweden (SW7388, female, 76 years old and SW510926, male, 51 years old) from 3-mm forearm dermal biopsies following informed consent under protocols approved by the Broad Institute, Harvard University, and Umea University.

## Supplementary Material

1

2

## Highlights

- Coupling NGN2 expression and SMAD/WNT inhibition yields human patterned induced neurons (hpiNs)
- Single-cell analysis indicates excitatory, neuronal identity, with variable maturity
- A CAMK2A::GFP reporter gene isolates more differentiated neurons from progenitors
- CAMK2A+ hpiNs display AMPAR- and NMDAR-mediated synaptic transmission
