# Supplementary material for: Combining NGN2 Programming with Developmental Patterning Generates Human Excitatory Neurons with NMDAR-Mediated Synaptic Transmission
Source: Cell Rep. Author manuscript; Available in PMC 2018 Jun 15. (PMC6003669; doi:10.1016/j.celrep.2018.04.066)
Supplement: 1 [file NIHMS972486-supplement-1.pdf]

Cell Reports, Volume *23*

Supplemental Information

Combining *NGN2* Programming with Developmental Patterning Generates Human Excitatory Neurons with NMDAR-Mediated Synaptic Transmission

Ralda Nehme, Emanuela Zuccaro, Sulagna Dia Ghosh, Chenchen Li, John L. Sherwood, Olli Pietilainen, Lindy E. Barrett, Francesco Limone, Kathleen A. Worringer, Sravya Kommineni, Ying Zang, Davide Cacchiarelli, Alex Meissner, Rolf Adolfsson, Stephen Haggarty, Jon Madison, Matthias Muller, Paola Arlotta, Zhanyan Fu, Guoping Feng, and Kevin Eggan

Inventory of Supplemental Information

Figure S1: Differentiation of hpiNs over time in culture. Related to Figure 1.

Figure S2: Reproducibility across independent lines and effect of small molecule patterning. Related to Figure 1 and 2.

Figure S3: Expression of CAMK2A, AMPAR and NMDAR subunits. Related to Figure 2.

Figure S4: Network activity of hpiNs over time and across lines. Related to Figure 3.

Figure S5: Electrophysiological properties of hpiNs and CAMK2A+ hpiNs over time. Related to Figure 4

Table S1. Ruling out non-forbrain identities of the hpiNs. Related to Figure 1.

Table S2. Fluidigm probes. Related to Figure 1.

Table S3. Categories and genes for density plot in Figure 1E. Related to Figure 1.

Table S4. Reproducible membrane properties. Comparison of membrane properties of neurons derived from three independent lines and three separate cultures batches. Related to Figure 4.

Table S5. Summary of assays with cells, samples and replicate numbers. Related to all Figures.

Supplemental Experimental Procedures. Methods for: hPCS lines and characterization, Human pluripotent stem cell culture, Infection of hPSCs with lentiviruses, immunocytochemistry, qPCR, Single Cell Expression Profiling, Total population RNASeq and Bioinformatic analysis, Brainspan analysis, Electrophysiological Studies, MEA. Related to method section.

Supplemental References.

Supplemental Figure S1

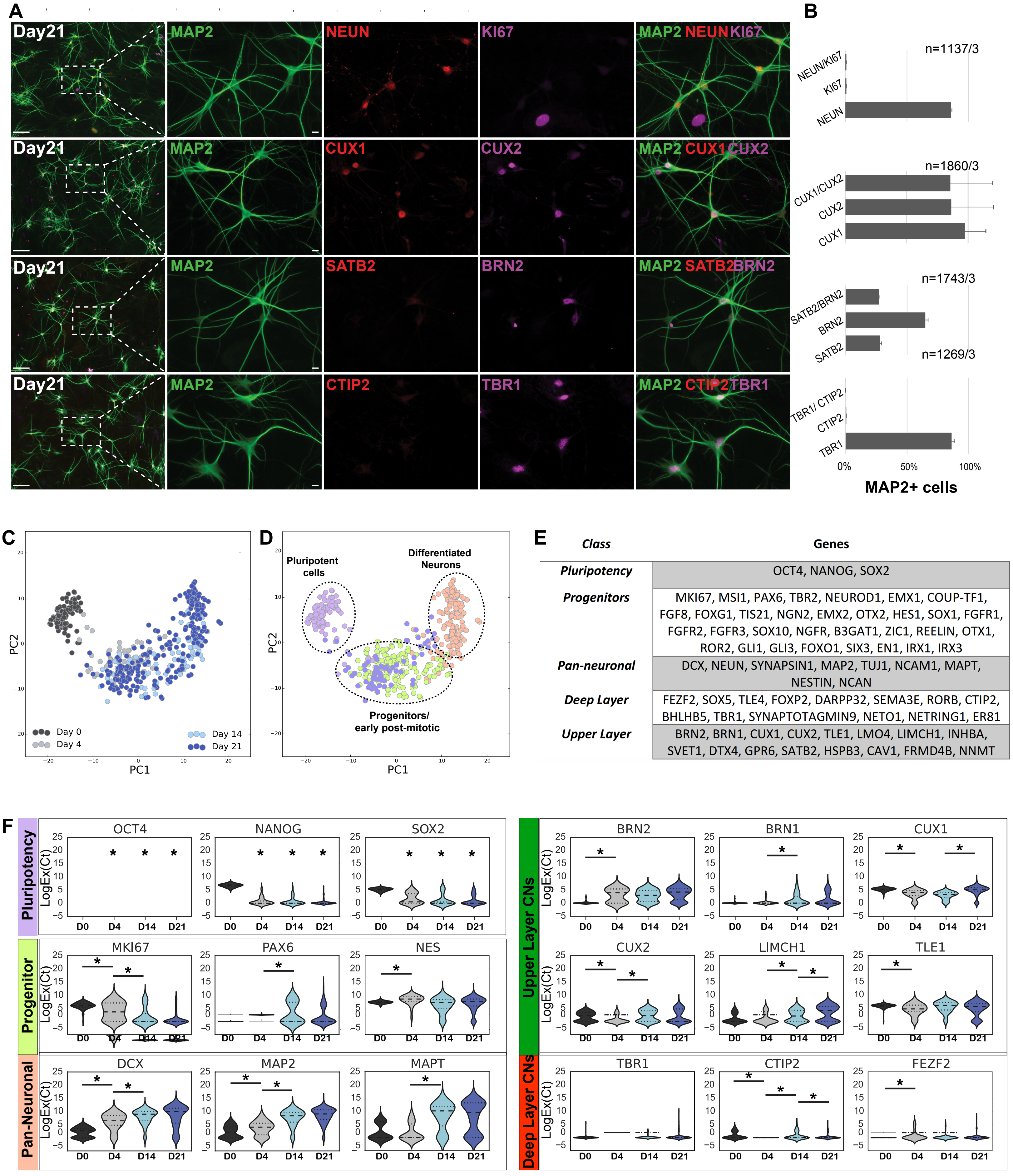

Figure S1. Differentiation of hpiNs over time in culture. Related to Figure 1. A) IF of neurons at 21 days. 20x and 40x images. B) Quantification of ICC. Using MAP2 expression to normalize quantification, we found that 95% of day21 neurons express NEUN while the proliferative marker KI67 was detected in only 1% of cells (n=1137 cells), the vast majority of day 21 hpiNs expressed CUX1 and CUX2, transcription factors that in mouse cortex are expressed in layer II-III (Tasic et al. 2016): CUX1 98% CUX2 91% CUX1/CUX2 co-expression 90% (n=1860 cells). BRN2, another marker of upper cortical layers in mouse, and one of the original neuronal conversion factors (Vierbuchen et al. 2010) was present in 50% of the hpiN day 21 population (n=1743 cells). By contrast, transcription factors that specify neurons in deep cortical layers, such as CTIP2, were virtually absent. Finally, 86% of the cells expressed TBR1, a transcription factor widely expressed in glutamatergic neurons (n=1269 cells). C) PCA plot of single cell Fluidigm Biomark gene expression data at day 0, 4, 14 and 21, (93 cells at d0, 51 cells at d4, 76 cells at d14, 239 cells at d21) (Chip1). D) Hierarchical Clustering. Three main clusters were observed. E) Subset of gene categories included in the Biomark Chip1. F) Violin plots of key genes.

Supplemental Figure S2

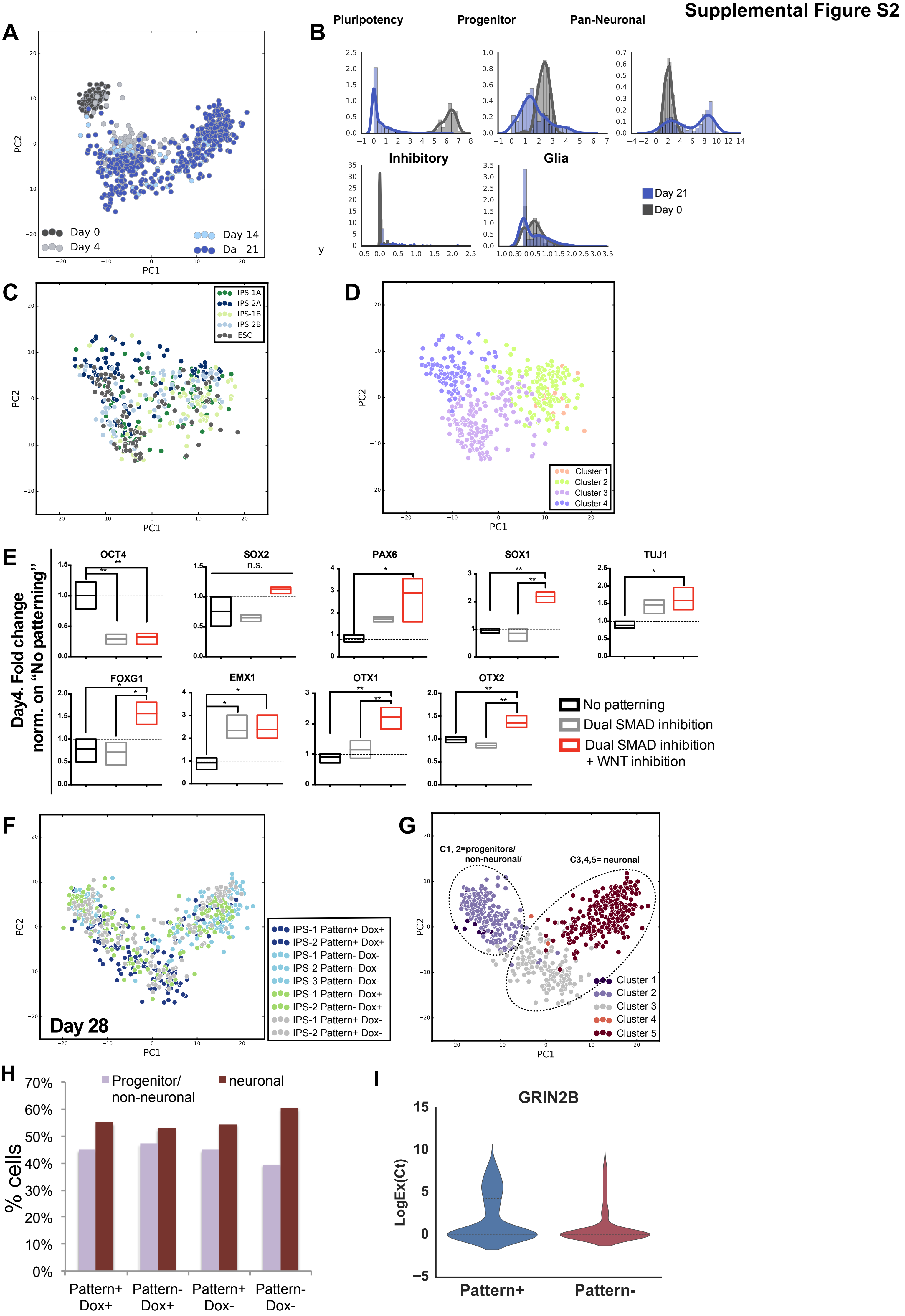

Figure S2. Reproducibility across independent lines and effect of small molecule patterning. Related to Figure 1. A) HC and B) density plots for iPS2 of single cell Fluidigm Biomark gene expression data over 21 days of differentiation. C) PCA and D) HC from different replicates of three iPS and ES1 lines at d21 showing reproducibility.E) qRT-PCR of day 4 cells that have been subjected to either no small molecule treatment (no patterning), dual SMAD inhibition (addition of SB431542 and LDN193189), or dual SMAD and WNT inhibition (addition of SB431542, LDN193189 and XAV939) in conjunction with NGN2 overexpression. F) PCA and G) HC of day 28 cells from three independent lines grown with alterations in the protocol (Pattern+, patterning with the small molecules, Pattern-, no patterning, Dox+, doxycycline treatment thought-out culture time, Dox-, doxycycline treatment for the first 4 days of culture). H) Quantification of the percentages of cells in each condition belonging to the neuronal or the progenitor/non-neuronal clusters. I) Violin plots showing the expression of GRIN2B in hpiNs at day21 in the presence or absence of patterning (n=3 differentiations, 3 lines; p value = 1.28e-07).

Supplemental Figure S3

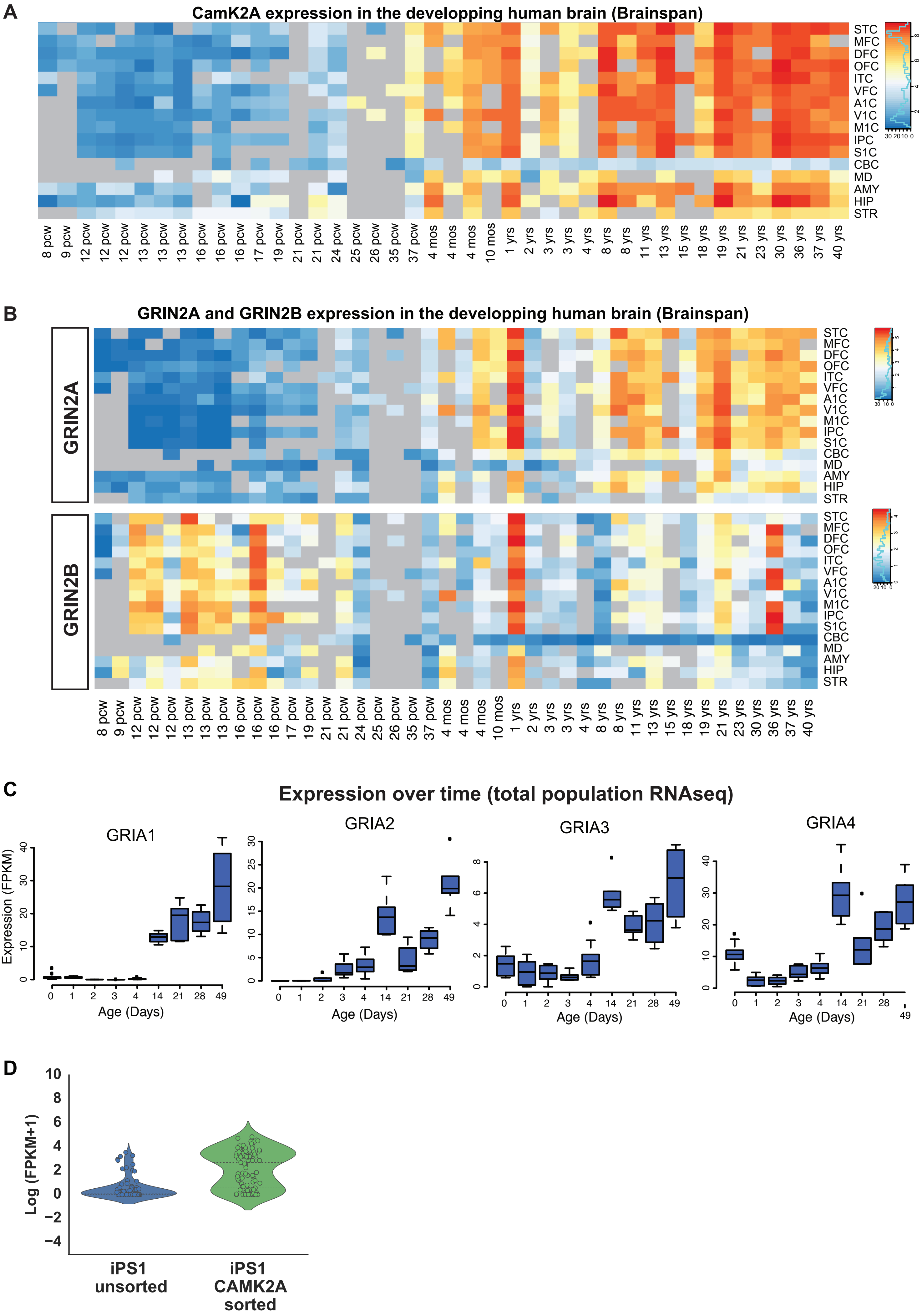

Figure S3. Expression of CAMK2A, AMPAR and NMDAR subunits. Related to Figure 2. A) Expression of CAMK2A in the developing human brain (Brainspan data). B) Expression of GRIN2A and GRIN2A in the developing human brain (Brainspan data). C) Expression of the AMPA receptor subunits in hpiNs over time (day 0 to day 49). D) Dispersion of the FPKM values for endogenous CAMK2A mRNA in CAMK2A+ cells.

Supplemental Figure S4

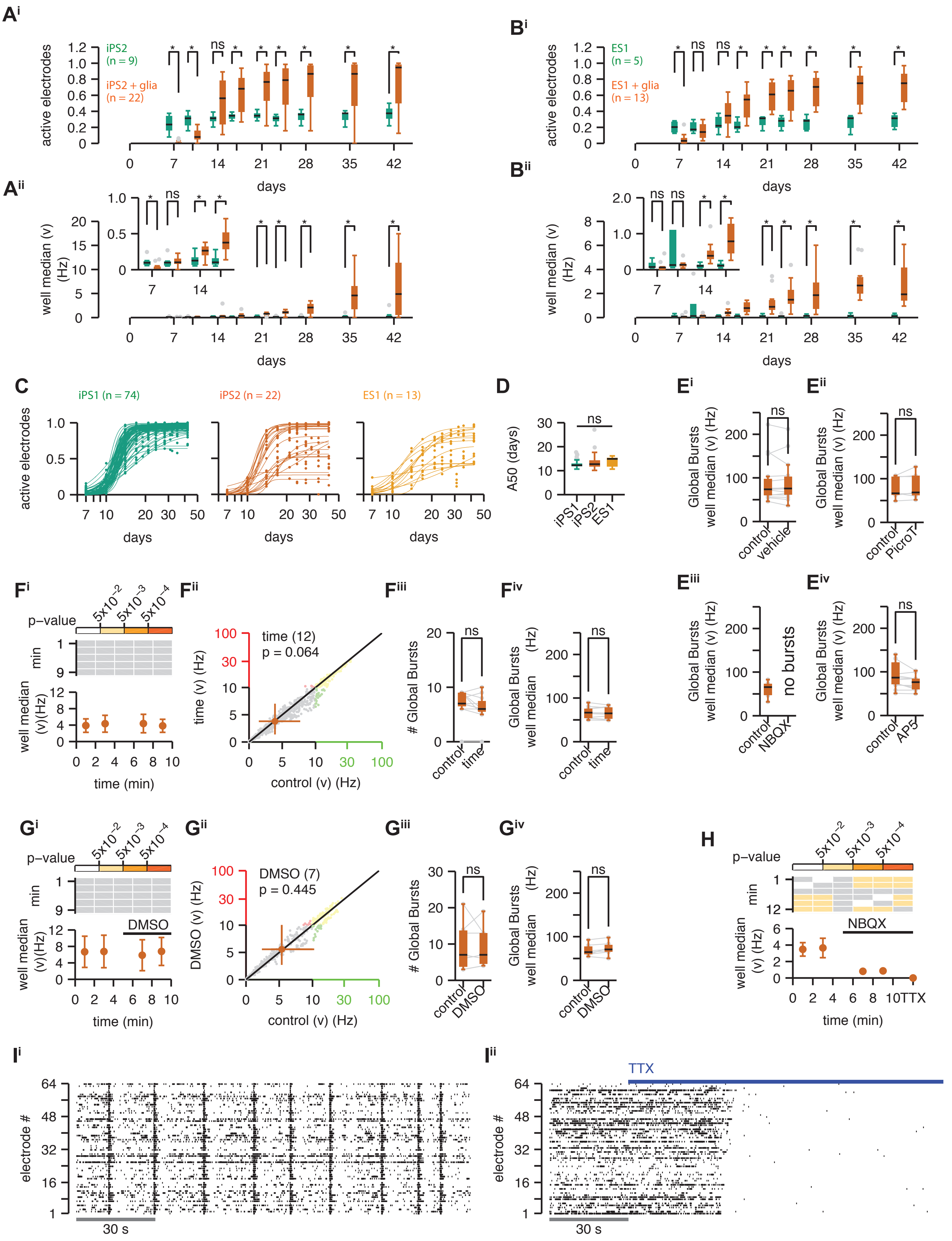

Figure S4. Network activity of hpiNs over time and across lines. Related to Figure 4. A) Comparison of neurons differentiated from iPS2 and B) from ES1 cultured with glia (green) and without glia (orange). C) Comparison of neurons derived from iPS1, iPS2 and ES1 cultured with glia. D) Comparison of A50 values from the three lines. E-I) Pharmacology analysis. F) Spike rate in time base control group. G) Picrotoxin Vehicle (DMSO) data. H) Quantification of TTX effect. I) Raster plot illustrating the effect of TTX on neuronal spiking.

Supplemental Figure S5

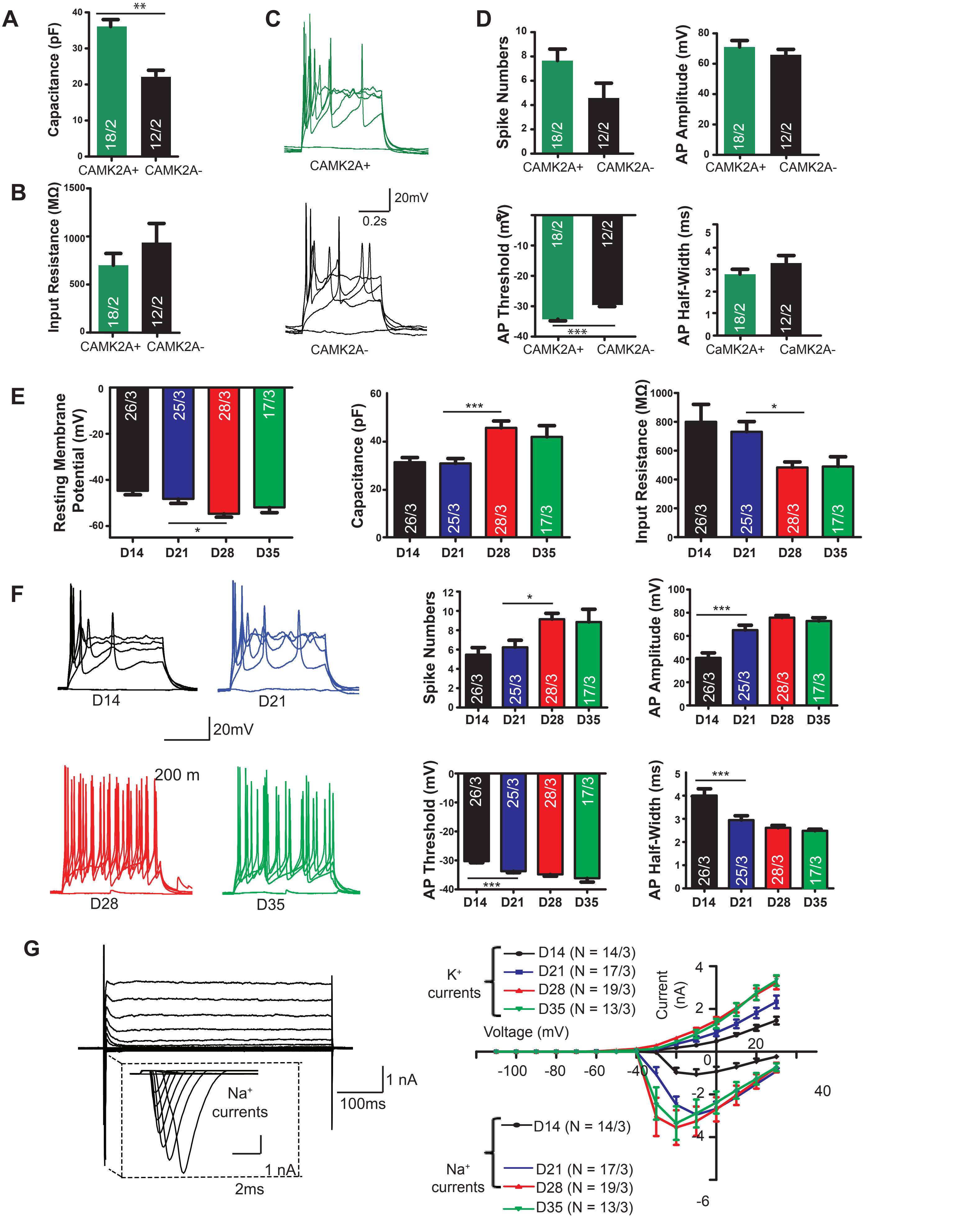

Figure S5. Electrophysiological properties of hpiNs and CAMK2A+ hpiNs over time. Related to Figure 5. A) the membrane capacitance of CAMK2A –GFP+ neurons was significantly larger than that of GFP negative cells. B) No significant change was detected in the membrane input resistance. C) Representative AP traces in response to step current injection of 20pA in current clamp mode for a CAMK2A –GFP+ cell and a GFP- cell at d21. D) Comparisons of average spike numbers/pulse, AP amplitude, AP threshold and AP half-width, respectively. Data are means ± SEMs. *p<0.01; **p<0.005; ***p<0.001, unpaired student t-test. E) CAMK2A-GFP+ neurons showed the average membrane potential significantly more hyperpolarized over time, the membrane capacitance was significantly higher at day 28, and the membrane input resistance decreased over time. F) Representative AP traces in response to step current injection of 20pA in current clamp mode for a cell at four different time point post-differentiation. Summary of average spike numbers/pulse, AP amplitude, AP threshold and AP half-width, respectively. G) AP firing properties consistently showed the time-dependent maturation. Numbers of cells / cultures are shown inside the bar. Data are means ± SEMs. *p<0.01; **p<0.005; ***p<0.001, unpaired student t-test.

Table S1. Ruling out non-forbrain identities of the hpiN. Related to Figure 1. Expression values of the listed genes are shown in cells collected at different time points during the differentiation scheme and subjected to population RNA sequencing

		**FPKM values**					
	**Gene**	**day0**	**day4**	**day14**	**day21**	**day28**	**day49**
motor neurons/ spinal identity	*MNX1*	0.07	0.12	0.18	0.09	0.11	0.11
	HOXA3	0.08	0.00	0.13	0.37	0.14	0.14
	HOXA4	0.00	0.00	0.00	0.06	0.01	0.01
	HOXA5	0.00	0.00	0.00	0.17	0.04	0.04
	HOX6	0.00	0.00	0.00	0.00	0.00	0.00
	HOX7	0.00	0.01	0.20	0.02	0.06	0.06
	HOXB7	0.00	0.07	0.00	0.04	0.03	0.03
	HOXB8	0.00	0.00	0.12	0.02	0.03	0.03
	HOXC4	0.00	0.02	0.30	1.45	0.44	0.44
	HOXC5	0.00	0.00	0.00	0.06	0.02	0.02
Hindbrain (Di Bonito et al. 2013)	*MAP1A*	1.02	12.57	10.03	14.83	9.61	9.61
	HOXA2	0.00	0.00	1.36	2.45	0.95	0.95
	GABBR2	0.52	2.83	3.16	2.14	2.16	2.16
	SLC1A6	5.45	5.35	11.71	5.03	6.88	6.88
MGE/ inhibitory neurons (Maroof et al. 2013)	*DLX2*	0.01	0.05	0.22	0.45	0.18	0.18
	DLX5	0.00	0.00	0.13	0.39	0.13	0.13
	NKX2.1	0.00	0.01	0.02	0.25	0.07	0.07
	LHX6	1.31	0.27	0.21	0.10	0.47	0.47
	GAD1	0.24	0.35	1.33	0.86	0.70	0.70
Hypothalamus (Merkle et al. 2015)	*RAX*	0.03	0.01	0.01	0.02	0.00	0.02
	OTP	0.00	0.01	0.09	0.07	0.24	0.33
	SIM1	0.69	26.34	8.34	2.18	2.20	2.19
Hippocampus (Yu et al. 2014)	*PROX1*	0.67	6.85	5.76	3.79	5.70	6.96
Diencephalus	*IRX1*	0.25	0.61	2.12	4.79	1.94	1.94
dopaminergic neuron/pons /medulla/ cerebellum (Kriks et al. 2011)	*NR4A2*	0.07	0.20	0.24	0.42	0.23	0.23
	SLC18A2	2.60	0.36	0.11	0.08	0.79	0.79
	LMX1A	0.00	0.16	2.18	2.97	1.33	1.33
	FOXA2	0.19	2.61	0.22	0.21	0.81	0.81

Table S2. Fluidigm probes. Related to Figure 1.

Table S3. Categories and genes for density plot in Figure 1E. Related to Figure 1.

Pluripotency	Progenitors (Forebrain)	Inhibitory progenitors	Pan Neuronal	Glial cells
Sox2	Pax6a	Dlx1	NeuN	PDGFRa
Oct4	NeuroD1	Dlx5	Synapsin1	s100B
Nanog	Sox2	Lhx8	DCX	olig2
Mki67	Emx2	Lhx6	Map2	GFAP
	Otx2	Nkx2.1	Tuj1	GSX2
	Hes1	Nkx6.2	NCAM	ALDH1l1
	Sox1	Nkx2.2	Mapt/Tau	NG2
	NCAN	Ascl1		
	MSI1			
	Mki67			

Table S4. Reproducible membrane properties. Related to Figure 4. Comparison of membrane properties of neurons derived from three independent lines and three separate cultures batches.

Cell lines	Cm (pF)	Rin (MΩ)	RMP (-mV)	Spikes N/pulse	AP Height (mV)	AP Threshold (-mV)	AP Half-Width (ms)	n (cells/ batches)
iPS1	46.25 ± 2.20	483 ± 37	55.25 ± 1.30	8.54 ± 0.59	75.79 ± 1.94	34.83 ± 0.63	2.62 ± 0.11	25/3
iPS2	30.47 ± 2.34	790 ± 73	43.13 ± 2.17	6.83 ± 1.28	66.47 ± 2.05	31.03 ± 0.74	2.81± 0.18	16/2
ES1	30.76 ± 2.37	725 ± 62	45.04 ± 0.88	7.00 ± 0.96	67.98 ± 1.96	32.64 ± 0.94	2.79 ± 0.10	19/2
iPS1-1	50.80± 4.22	438 ± 63	55.62 ± 1.84	8.86 ± 0.56	74.04 ± 1.83	34.55 ± 0.65	2.44 ± 0.08	8/1
iPS1-2	44.75 ± 3.96	485 ± 68	52.08 ± 3.42	7.75 ± 0.73	78.25 ± 4.35	35.40 ± 1.32	2.87 ± 0.05	8/1
iPS1-3	42.33± 1.64	534 ± 58	58.68 ± 1.69	9.20 ± 1.49	77.06 ± 3.08	34.93 ± 1.30	2.72 ± 0.28	9/1

Table S5. Summary of assays with cell, sample and replicate numbers. Related to all Figures.

Assay	Days *in vitro*	iPS1	iPS2	ES1	iPS3
**Fluidigm Chip 1**	0	n=93 cells	n=94 cells		
	4	n=51 cells	n=82 cells		
	14	n=76 cells	n=84 cells		
	21	n=239 cells	n=400 cells		
**Fluidigm Chip 2**	14	n=88 cells	n=88 cells		
	21	n=264 cells	n=263 cells		
	28	n=161 cells, n=81 cells*	n=74 cells	n= 92 cells	n=73 cells
**Single cell RNAseq**	28	n=73 cells, n=93 cells*			
**Population RNAseq**	0	n=3 replicates	n=3 replicates		
	1	n=3 replicates	n=3 replicates		
	2	n=3 replicates	n=3 replicates		
	3	n=3 replicates	n=3 replicates		
	4	n=3 replicates	n=3 replicates		
	14	n=3 replicates	n=3 replicates		
	21	n=3 replicates	n=3 replicates		
	28	n=3 replicates	n=3 replicates		
	49	n=3 replicates	n=3 replicates		
**MEA**	7	No Glia n=15 wells (3 replicates)<br>With Glia n=74 wells (6 replicates)	No Glia n=9 wells (2 replicates)<br>With Glia n=22 wells (3 replicates)	No Glia n=5 wells (1 replicates)	
	10	No Glia n=15 wells (3 replicates)<br>With Glia n=70 wells (5 replicates)	No Glia n=9 wells (2 replicates)<br>With Glia n=22 wells (3 replicates)	No Glia n=5 wells (1 replicates)	
	14	No Glia n=15 wells (3 replicates)<br>With Glia n=74 wells (6 replicates)	No Glia n=9 wells (2 replicates)<br>With Glia n=22 wells (3 replicates)	No Glia n=5 wells (1 replicates)	
	17	No Glia n=9 wells (3 replicates)<br>With Glia n=28 wells (4 replicates)	No Glia n=9 wells (2 replicates)<br>With Glia n=22 wells (3 replicates)	No Glia n=5 wells (1 replicates)	
	21	No Glia n=15 wells (3 replicates)<br>With Glia n=74 wells (6 replicates)	No Glia n=9 wells (2 replicates)<br>With Glia n=22 wells (3 replicates)	No Glia n=5 wells (1 replicates)	
	24	No Glia n=15 wells (3 replicates)<br>With Glia n=74 wells (6 replicates)	No Glia n=9 wells (2 replicates)<br>With Glia n=22 wells (3 replicates)	No Glia n=5 wells (1 replicates)	
	28	No Glia n=15 wells (3 replicates)<br>With Glia n=74 wells (6 replicates)	No Glia n=9 wells (2 replicates)<br>With Glia n=22 wells (3 replicates)	No Glia n=5 wells (1 replicates)	
	35	No Glia n=15 wells (3 replicates)<br>With Glia n=74 wells (6 replicates)	No Glia n=9 wells (2 replicates)<br>With Glia n=22 wells (3 replicates)	No Glia n=5 wells (1 replicates)	
	42	No Glia n=15 wells (3 replicates)<br>With Glia n=74 wells (6 replicates)	No Glia n=9 wells (2 replicates)<br>With Glia n=22 wells (3 replicates)	No Glia n=5 wells (1 replicates)	
**Whole-Cell Patch Clamp**	14	n=32 cells (2 replicates), n=26 cells (3 replicates)*			
	21	n=10 cells (2 replicates), n=25 cells (3 replicates)*			
	28	n=10 cells (2 replicates), n=28 cells (3 replicates)*	n=16 cells (2 replicates)*	n=19 cells (2 replicates)*	
	35	n=10 cells (2 replicates), n=17 cells (3 replicates)*			

*=CAMK2A-GFP counts

Supplemental Experimental Procedures

hPCS lines and characterization
Human iPSC lines SW7388-1 (iPS1) and SW510926-11 (iPS2) were generated from fibroblasts using the non-integrating CytoTune™ - Sendai viral vector kit (Life Technologies, A13780), which includes the reprogramming factors Oct3/4, Sox2, Klf4 and c-Myc. Fibroblasts from these two lines were collected in Umea, Sweden, and generated from 3mm forearm dermal biopsies following informed consent under protocols approved by the Broad Institute, Harvard University and Umea University. One to two weeks later, fibroblast outgrowths from the explants were passaged with trypsin and frozen. The NanoString nCounter human karyotype panel (NanoString Technologies) was used per manufacturer's instructions to evaluate cell lines for large-scale CNVs. Sample counts were normalized to a reference sample with a normal karyotype. Hues63 was obtained from the Human Embryonic Stem Cell Facility of the Harvard Stem Cell Institute. iPS hDFn 83/22 iNgn2#9 (iPS3) was generated as previously described (Bidinosti et al. 2016).

Human pluripotent stem cell culture
Human ESCs and iPSCs were maintained on plates coated with geltrex (life technologies, A1413301) in mTeSR media (Invitrogen, Calsbad, CA) and passaged with accutase (Gibco, A11105). All cell cultures were maintained at 37°C, 5% CO2.

Infection of hPSCs with lentiviruses
Lentivirus particles were produced by Alstem (http://www.alstembio.com/). hPSCs were seeded in a geltrex coated 12 well plate at a density of 100,000 cells/cm2 in mTeSR supplemented with rock inhibitor. The next day, cells were fed with lentivirus infection medium (lentiviruses in mTeSR media), and infection was continued for 24 hr.

Immunocytochemistry
For immunocytochemistry, cells were fixed in ice cold 4% paraformaldehyde for 20min at room temperature and then permeabilized with 0.3% TritonX at room temperature. Cells were then blocked in 3% BSA in PBS for 1 hour at room temperature. Primary antibodies were incubated at 4C overnight in blocking buffer. Secondary antibodies were incubated for 2 hours at room temperature in blocking buffer. Primary antibodies used and their respective dilutions were: Goat anti-Nanog (R&D, AF1997 -1:300), Rat anti-Oct4 (R&D, MAB1759 -1:300), Chicken anti-Map2 (Abcam, ab5392 -1:10000), Rabbit anti-Tbr1 (Abcam, ab31940 -1:2000), Rabbit anti-Ki67 (BD, 550609 -1:300), Mouse anti-NeuN (1:300), Mouse anti-Satb2 (Abcam, ab51502 -1:50), Rat anti-Ctip2 (Abcam, ab18465 -1:100), Rabbit anti-Brn2 (Abcam, ab137469 -1:300), Mouse anti-CutL1 (Abcam, ab54583 -1:300). All secondary antibodies were used at 1:750 dilutions (Alexa 488/546/647).

qPCR
RNA was extracted from three replicate wells per condition with the miRNeasy Micro Kit (Qiagen). cDNA was produced with iScript kit (BioRad) using 300 ng of RNA. RT-qPCR reactions were performed in triplicates using 3 μl of cDNA with SYBR Green

(BioRad) and were run on a CFX96 Touch™ PCR Machine for 39 cycles at: 95°C for 15s, 60°C for 30s, 55°C for 30s. Differences in gene expression levels were determined by comparing each sample to the no-patterning condition after normalization to GAPDH.

Single Cell Expression Profiling

Single Cell qPCR was performed using Biomark Real-Time PCR Analysis version 4.0 (Fluidigm) as per manufacturer's instructions by the Broad Genomics Platform. Raw Ct values were transformed to relative expression levels (Log2Ex) by subtracting a threshold value of 22 or the minimum value detected for a given gene across all experiments if less than 22. Cells with weak or no expression were defined as those expressing only one of three house keeping genes or cells expressing less than 10 genes and were removed from analysis. Data normalization was performed by first calculating the geometric mean of house keeping genes (HKgm) for each cell and dividing it by the median of geometric means for each house keeping gene (HKGMmedian) to generate a normalization factor (NFcell = HKgm/ HKGMmedian). Expression values for each cell were multiplied by NFcell to generate a normalized dataset. Analysis of data and visualization of results were performed using Python packages (Numpy 1.7.1, Pandas 0.12.0, SciPy 0.12.0, Matplotlib 1.2.1) and R statistical software (3.2.3). To identify cells with similar gene expression profiles and genes contributing to variation across cells we used hierarchical clustering (HC) and principal component analysis (PCA). Hierarchical clustering was performed on all cells using Euclidian distance metric and complete linkage (Python package fastcluster 1.1.20). PCA was performed using the decomposition.PCA module from the scikit-learn Python package (version 0.14). Since single cell gene expression data is zero-inflated and violates assumptions of normality, we applied nonparametric statistical approaches to test for significant differences in gene expression between cell populations. Normalized expression levels between populations were compared using either the Mann-Whitney test or the Kruskal-Wallis test with Bonferonni correction to account for multiple comparisons. Single cell RNA sequencing libraries were prepared according to the SmartSeq2 protocol (Picelli et al. 2014) with minor modifications (Trombetta et al. 2014). All libraries were prepared by the Broad Technology Labs and sequenced by the Broad Genomics Platform. Briefly, total RNA was purified using RNA-SPRI beads. Poly(A)+ mRNA was converted to cDNA which was then amplified. cDNA was subject to transposon-based fragmentation that used dual-indexing to barcode each fragment of each converted transcript with a combination of barcodes specific to each sample. Each cell was given its own combination of barcodes. Barcoded cDNA fragments were then pooled prior to sequencing. Sequencing was carried out as paired-end 2x25bp with an additional 8 cycles for each index. To obtain expression values for each cell, the data was separated by barcode and aligned using Tophat version 2.0.10 (Kim et al. 2013) with default settings. Transcripts were quantified by the BTL computational pipeline using Cuffquant version 2.2.1 (Trapnell et al. 2012). Expression levels were converted to log-space by taking the log2(FPKM +1).

Total population RNASeq and Bioinformatic analysis

Cells were harvested in RTLplus Lysis buffer (Quiagen 1053393) and stored at -80°C. Sequencing libraries were generated from 100 ng of total RNA using the TruSeq RNA Sample Preparation kit (Illumina RS-122-2303) and quantified using the Qubit

fluorometer (Life Technologies) following the manufacturer's instructions. Libraries were then pooled and sequenced by high output run on a HiSeq 2500 (Illumina). The total population RNA-seq fastq data was aligned against ENSEMBL human reference genome (build GRCh37/hg19) using STAR (v.2.4.0) (Dobin et al. 2013). Cufflinks (v.2.2.1) (Trapnell et al. 2012) was used to derive normalized gene expression in fragments per kilo base per million (FPKM). The following analysis only included genes that had an average expression of more than 1 FPKM (N (genes)=17922). Principal component analysis (PCA) was performed in R with prcomp. The PCA was run using regularized log transformation of read counts for 500 genes with the highest variance in expression among the stem cell derived neurons. The read counts were obtained from the aligned BAM-files in R using Rsubread (Liao et al. 2014) and further transformed using DESeq2 (Love et al. 2014) prior to PCA.

Brainspan analysis
The RPKM normalized expression data for 572 postmortem brain samples was downloaded from the BrainSpan project (http://www.brainspan.org). The present analysis included RNA-Seq data from five cortical brain regions obtained from different stages of developing brain ranging from 8 weeks post conception to 40 years. The cortical brain regions included anterior (rostral) cingulate (medial prefrontal) cortex (MFC), dorsolateral prefrontal cortex (DFC), orbital frontal cortex (OFC), inferolateral temporal cortex (ITC), ventrolateral prefrontal cortex (VFC). Genes that had a standard deviation less than or equal to 1 were removed from the analysis to reduce redundant information, leaving us with expression data from 16073 genes in 167 samples from the developing brain. A Pearson correlation was calculated between the log2(RPKM+1) values from the five cortical regions from BrainSpan at different ages and the log2(FPKM+1) values from different time points of the stem cell derived neurons. The correlation analysis included 12119 genes that were unequivocally named and passed quality control in the two data sets. An average of the correlations was calculated within each time point to compress the 167×64 correlation matrix to 167×9 matrix.

Electrophysiological Studies
Whole-cell patch clamp recordings were performed on cultured human induced iPSCs as described (Zhang et al 2013). Recording pipettes (KG33, King Precision Glass) were pulled in a horizontal pipette puller (P-97, Sutter Instruments) with a tip resistance of 3–5 MΩ. All experiments were performed at room temperature unless otherwise specified. For the whole-cell recording of voltage dependent Na+ and K+ currents, pipettes were filled with the internal solution containing (in mM): 131 K-gluconate, 17.5 KCl, 9 NaCl, 1 MgCl2.6H2O, 10 HEPES, 1.1 EGTA, 2 ATP magnesium salt, and 0.2 GTP sodium salt. pH was adjusted to 7.2-7.4 with KOH, and osmolarity was adjusted to 298–300mOsm with sucrose. In current-clamp mode, membrane potential was held at −65 mV with a multiclamp 700B amplifier (Molecular Devices, Sunnyvale, CA, USA), and step currents were then injected to elicit action potentials. For AMPA receptor mediated excitatory postsynaptic currents (EPSCs), pipettes were filled with the internal solution containing (in mM): 110 CsOH (50% wt), 110 D-Gluconic acid (49-53% wt), 4 NaCl, 15 KCl, 5 TEA-Cl, 20 HEPES, 0.2 EGTA, 5 lidocaine N-ethyl chloride, 4 ATP magnesium salt, and 0.3 GTP sodium salt. pH was adjusted to 7.2-7.4 with CsOH, and osmolarity

was adjusted to 298–300mOsm with about 10 mM K_2SO_4. Cultured cells were constantly perfused at a speed of 3ml/min with an extracellular solution containing (in mM): 119 NaCl, 2.3 KCl, 2 $CaCl_2$, 1 $MgCl_2$, 15 HEPES, 5 glucose, phenol red (0.25mg/L) and D-serine (10μM) (all from sigma) adjusted to pH 7.2-7.4 with NaOH. Osmolality was adjusted to 325 mOsm with sucrose. The spontaneous synaptic events were not recorded until 5 min after entering whole-cell patch clamp recording mode to allow the dialysis of Cs^+ internal solution for a relatively complete block of the potassium channels in the neurons recorded. Cells in which the series resistance (Rs, typically 8–12 MΩ) changed by >20% were excluded for data analysis. In addition, cells with Rs more than 20 MΩ at any time during the recordings were discarded. Evoked synaptic responses were triggered by 1 ms current injection through a local concentric bipolar stimulating electrode (CBARC75, FHC, USA) placed about 100–150μm from the soma of neurons recorded. Picrotoxin (Sigma, 50 μM) was used to block inhibitory synaptic transmissions. NBQX (Sigma) and DL-APV (Sigma, 25 μM) were used to inhibit AMPA receptor mediated and NMDA receptor mediated excitatory synaptic responses, respectively. Whole-cell NMDA-evoked current response was triggered by pressure injection of 100 μM NMDA in Mg^{2+} free external solution with 50 μM Picrotoxin, 10 μM NBQX and 1 μM TTX with a picospritzer (10 psi; 200ms duration, Picospritzer III, General Valve, Fairfield, NJ). All whole-cell recording data were sampled at 10 kHz with a 2 kHz low-pass filter with a Digidata 1440A (Molecular Devices). All data were stored in a computer for subsequent off-line analysis. Spontaneous and miniature EPSCs were detected and analyzed with MiniAnalysis (Synaptosoft, Decatur, Georgia, USA). Biocytin 0.5% was added to the recording pipettes in some experiments. After filling cells for 10 minutes, slides were fixed overnight with 4% paraformaldehide in 0.1 M PBS, pH 7.4. Slides were washed in PBS and incubated in 20% normal BSA. Primary antibody to GFP (Chicken monoclonal 1:1000, Millipore, Darmstadt, Germany) was incubated overnight at 4ºC and stained with secondary goat antibody conjugated to Alexa 488 (1:200). At least 2 h were allowed for binding before rinsing in PBS. Additionally, the slides were incubated 2 h in PBS solution containing 0.2 Triton X-100 and streptavidin conjugated to Alexa 568(6 μl/ml; Abcam). A spinning disc confocal microscope (Nikon NC2) was used and pictures were taken using NIS software.

MEA: Materials

Extracellular spikes (action potentials) acquired using Axion Biosystems multi-well micro-electrode array (MEA) plate system (The Maestro, Axion Biosystems; 64 electrodes per culture well). During the recording period, the plate temperature was maintained at 37± 0.1 °C, environmental gas composition was not maintained outside of the incubator.

Data acquisition and analysis

To prevent aliasing, and facilitate spike detection, analog signals were band pass filtered at 0.2 - 3 kHz (Butterworth filters), and sampled at 12.5 kHz. Spike detection threshold set to 5 x root mean square (rms) of the electrode noise (to prevent biases, rms was calculated from periods without action potentials and fixed for the recording session). Neuronal activity is summarized by binning spikes (10 second bins), and reporting the average firing rate over K consecutive trials. More formally, if spike

count k in interval of duration T is given by n_k . The mean spike count ⟨n⟩ is calculated by dividing the sum of n_k by K (equation 1); and the mean spike rate ⟨ν⟩ by dividing ⟨n⟩ b y T (equation 2).

$$\langle n \rangle = \frac{1}{K}\sum_{k=1}^{K} n_k \quad (equation\ 1)$$

$$\langle \nu \rangle = \frac{\langle n \rangle}{T} \quad (equation\ 2)$$

Bursts have been identified by algorithms described by Wagenarr et al. (Wagenaar 2005). In brief, single channel bursts occur when at least four spikes occur at 4 times the channels mean firing rate (Wagnenarr's burstlets). A network burst is defined by temporally overlapping burstlets. A global burst is identified when at least 70% of the active channels are involved in a network burst. All post-processing and data analysis was performed using MATLAB® (MathWorks), and custom algorithms developed in-house.

MEA: Drugs

The following drugs were used: picrotoxin (Abcam), D-(-)-2-Amino-5-phosphonopentanoic acid (D-AP5, Abcam), 2,3-Dioxo-6-nitro-1,2,3,4-tetrahydrobenzo[f]quinoxaline-7-sulfonamide disodium salt (NBQX disodium salt, Abcam), Tetrodotoxin citrate (TTX, Abcam). Stock solutions for D-AP5 (100 mM), NBQX (100 mM), and TTX (10 mM) were prepared in ddH20. Stock solutions of Picrotoxin (100 mM) was prepared in Dimethyl sulfoxide (DMSO, EMD Millipore). Prior to use, working solution(s) were prepared by diluting stock solution(s) in culture media; such that adding 5 µl of the working solution, to each MEA plate well, produced the desired final concentration.

MEA: Statistics

Unless otherwise stated, descriptive statistics for MEA data is presented as Tukey style box plots, showing the 1st, 2nd, and 3rd quantile (Q1, Q2, & Q3 respectively; inter-quartile range, IQR = Q3- Q1). Box plot whiskers extend to the most extreme data points between Q1-1.5*IQR and Q3+1.5*IQR (McGill 1978, Krzywinski and Altman 2014, Streit and Gehlenborg 2014). All data points outside the whiskers are plot. Non-parametric 95 % confidence intervals for M are calculated using fractional order statistics (Hutson 1999).

In each culture well the MEA chip records activity at 64 probe sites. Firing rates of neurons recorded from the same culture well are not independent, but clustered. The 6th minute, used to apply vehicle / drug, is omitted. To test for significant difference in the ⟨ν⟩ before and after pharmacological manipulations, were the ⟨ν⟩ pairs are clustered according to their culture well, a signed rank test for clustered data was applied (Datta and Satten 2008, Galbraith et al. 2010). Standard statistical methods are used for the comparisons of the culture well median ⟨ν⟩, were the use of summary statistics reduces the sampling clusters to single independent observations. For non-parametric data sets, the differences between two independent groups was assessed by a two-sided Wilcoxon rank sum test (5 % significance level). For >2 independent groups, the Kruskal-Wallis test by ranks was applied (5% significance level). When

considering repeated measures of ≥ 3 paired groups, the Freidman test was used to test the H0: sum of the ranks of the groups are the same (5 % critical level for rejecting H0 was taken from $\chi 2$ distribution). In the pharmacology studies performed at day 42, the culture well median $\langle \nu \rangle$ across MEA plate wells was normally distributed (these data are presented as mean ± s.d.). To test the H0: means are equal, a repeated measures analysis of variance was applied. If Mauchly's test for sphericity indicated that the assumption of sphericity had been violated (5% significance level), a Greenhouse-Geisser correction was applied to the repeated measures ANOVA. If the omnibus H0 was rejected at the 5% significance level, post-hoc tests were applied to identify which pairs of means are significantly different (Tukey's honest significant difference criterion, 5 % significance level).

Supplemental References

Arlotta, P., B. J. Molyneaux, J. Chen, J. Inoue, R. Kominami and J. D. Macklis (2005). "Neuronal subtype-specific genes that control corticospinal motor neuron development in vivo." Neuron **45**(2): 207-221.

Bidinosti, M., P. Botta, S. Kruttner, C. C. Proenca, N. Stoehr, M. Bernhard, I. Fruh, M. Mueller, D. Bonenfant, H. Voshol, et al. (2016). "CLK2 inhibition ameliorates autistic features associated with SHANK3 deficiency." Science **351**(6278): 1199-1203.

Datta, S. and G. A. Satten (2008). "A signed-rank test for clustered data." Biometrics **64**(2): 501-507.

Dobin, A., C. A. Davis, F. Schlesinger, J. Drenkow, C. Zaleski, S. Jha, P. Batut, M. Chaisson and T. R. Gingeras (2013). "STAR: ultrafast universal RNA-seq aligner." Bioinformatics **29**(1): 15-21.

Galbraith, S., J. A. Daniel and B. Vissel (2010). "A study of clustered data and approaches to its analysis." J Neurosci **30**(32): 10601-10608.

Hutson, A. D. (1999). "Calculating nonparametric confidence intervals for quantiles using fractional order statistics. ." Journal of Applied Statistics **26** 343-353.

Kim, D., G. Pertea, C. Trapnell, H. Pimentel, R. Kelley and S. L. Salzberg (2013). "TopHat2: accurate alignment of transcriptomes in the presence of insertions, deletions and gene fusions." Genome Biol **14**(4): R36.

Krzywinski, M. and N. Altman (2014). "Visualizing samples with box plots." Nat Methods **11**(2): 119-120.

Liao, Y., G. K. Smyth and W. Shi (2014). "featureCounts: an efficient general purpose program for assigning sequence reads to genomic features." Bioinformatics **30**(7): 923-930.

Love, M. I., W. Huber and S. Anders (2014). "Moderated estimation of fold change and dispersion for RNA-seq data with DESeq2." Genome Biol **15**(12): 550.

McGill, R., Tukey, J.W. and Larsen, W.A. (1978). "Variations of Box Plots." The American Statistician **32**: 12-16.

Picelli, S., O. R. Faridani, A. K. Bjorklund, G. Winberg, S. Sagasser and R. Sandberg (2014). "Full-length RNA-seq from single cells using Smart-seq2." Nat Protoc **9**(1): 171-181.

Streit, M. and N. Gehlenborg (2014). "Bar charts and box plots." Nat Methods **11**(2): 117.

Trapnell, C., A. Roberts, L. Goff, G. Pertea, D. Kim, D. R. Kelley, H. Pimentel, S. L. Salzberg, J. L. Rinn and L. Pachter (2012). "Differential gene and transcript expression analysis of RNA-seq experiments with TopHat and Cufflinks." Nat Protoc **7**(3): 562-578.

Trombetta, J. J., D. Gennert, D. Lu, R. Satija, A. K. Shalek and A. Regev (2014). "Preparation of Single-Cell RNA-Seq Libraries for Next Generation Sequencing." Curr Protoc Mol Biol **107**: 4 22 21-17.

Wagenaar, D. A., DeMarse, T.B. and Potter, S.M. (2005). MEABench: A toolset for multi-electrode data acquisition and on-line analysis. Conference Proceedings. 2nd International IEEE EMBS Conference on Neural Engineering, 2005., M. Akay, L. J. Wolf, and J. Stock, eds.
